# Global inequalities in arts, music or educational organization membership: an epidemiological analysis of 73,825 adults from 51 countries

**DOI:** 10.1186/s44263-025-00187-1

**Published:** 2025-08-08

**Authors:** Hei Wan Mak, Jessica K. Bone, Taiji Noguchi, Joeun Kim, Rina So, Emma Walker, Calum Smith, Marlee Bower, Ferdi Botha, Nisha Sajnani, Nils Fietje, Daisy Fancourt

**Affiliations:** 1 Department of Behavioural Science & Health, Institute of Epidemiology & Health Care, University College London, 1-19 Torrington Place, London, WC1E 7HB UK; 2 Department of Social Science, National Center for Geriatrics and Gerontology, Research Institute, 7-430 Morioka, Obu, Aichi 474-8511 Japan; 3 Japan Society for the Promotion of Science, 5-3-1 Kojimachi, Chiyoda, Tokyo 102-0083 Japan; 4 Korea Development Institute School of Public Policy and Management, 263 Namsejongro, Sejong, 30149 South Korea; 5 Department of Epidemiology & Public Health, Institute of Epidemiology & HealthCare, University College London, 1-19 Torrington Place, London, WC1E 7HB UK; 6 Nuffield Department of Population Health, University of Oxford, Oxford, OX3 7LF UK; 7 The Matilda Centre for Research in Mental Health and Substance Use, Faculty of Medicine and Health, The University of Sydney, Camperdown, NSW Australia; 8 Melbourne Institute: Applied Economic & Social Research, The University of Melbourne, Melbourne, VIC Australia; 9 Australian Research Council Centre of Excellence for Children and Families Over the Life Course, The University of Melbourne, Melbourne, VIC Australia; 10 Program in Drama Therapy, Jameel Arts & Health Lab, New York University, New York, NY USA; 11 World Health Organization Regional Office for Europe, Copenhagen, Denmark

**Keywords:** Arts and cultural engagement, Global inequalities, Multilevel modelling, World Values Survey

## Abstract

**Background:**

Arts and cultural engagement is a ubiquitous human behavior and considered to be evolutionarily and developmentally adaptive, with wide-ranging health benefits. Although levels of engagement are known to be far from equal within countries, comprehensive data comparing the rates and profiles of engagement on a global scale are lacking.

**Methods:**

This study analyzed cross-sectional data from Wave 7 of the World Values Survey (2017–2023), with 73,825 respondents from 51 countries. Using multilevel logistic regression, we tested whether a set of individual-level demographic and socio-economic factors and country-level socio-demographic and political factors were associated with arts, music, or educational organization membership rates. We additionally explored whether those individual-level factors differed across regions using single-level regression.

**Results:**

Prevalence of arts organization membership varies substantially across countries, from fewer than 1 in 30 people living in Egypt to more than 1 in 2 people living in Kenya. Education, income, and age are consistently identified individual-level factors contributing to membership disparities, alongside other region-specific individual-level factors such as gender and employment status. Membership is also positively influenced by country-level income inequality and net migration rate.

**Conclusions:**

We found a universal social gradient in membership, which suggests differential opportunities and potential barriers to access arts organizations across population subgroups. Given that participation in arts organizations has been shown to improve health outcomes, reducing disparities in access to and participation in the arts may have the potential to help reduce health inequalities and should therefore be a priority in global health.

**Supplementary Information:**

The online version contains supplementary material available at 10.1186/s44263-025-00187-1.

## Background

Arts and cultural engagement is a ubiquitous human behavior, found across history, cultures and societies globally, and is considered to be evolutionarily and developmentally adaptive [1]. It involves a broad range of art forms such as music, dance/movement, literary arts, visual arts, craft and design, theatre/performance, and media. These art forms can be engaged with in diverse ways, including attending cultural events and activities, creating or practising art, participating in cultural arts practices, learning in and through the arts, and consuming arts via various media [2]. A 2019 World Health Organization (WHO) Health Evidence Synthesis Report on the role of arts in health identified over 3000 studies internationally demonstrating the role of the arts in the prevention, management, and treatment of mental and physical illness as well as in broader health promotion [3]. Theories borrowing from pharmacological literature have highlighted that the arts contain multiple salutogenic ‘active ingredients’ [4], such as social interactions, cognitive stimulations, and mindfulness, which simultaneously activate various biological and psychological mechanisms to support health [5]. This has positioned the arts as a key modifiable health behavior, along with physical activity, dieting, substance use and sleep, and led to increasing global interest in how to enhance access to arts and cultural engagement [6].

However, despite the health benefits of the arts, levels of engagement in the arts are far from equal within countries. Comprehensive data on international cultural participation are lacking. In the past decade, however, there has been increasing interest in how to measure arts and cultural participation in a way that facilitates meaningful comparisons across different settings [7]. In 2015, Eurostat tracked arts and cultural participation (measured as going to the cinema, attending live performances, visiting cultural sites and practising artistic activities, e.g., playing a musical instrument, singing, dancing or painting) across over 30 European countries for the first time [8]. They showed stark differences in participation. For instance, less than 48% of people in South-Eastern Europe (such as the Balkans) had gone to the cinema, attended a live performance or visited a cultural site, compared to 80% or more amongst those living in Nordic countries, the Netherlands and Switzerland. Although this research only focused on European countries, it highlighted considerable disparities in arts and cultural engagement rates, even among nations with shared cultural, social, economic and political values. This raises the question of how participation rates differ between countries with diverse values and cultural settings.

Beyond understanding *rates* of participation, it is also important to understand and identify *profiles* of participation, i.e., who engages in arts and cultural activities. Research from sociology, economics, and epidemiology has highlighted that there is a social gradient in arts and cultural engagement, where people with higher educational attainment and from a more privileged background engage more in the arts (including visiting cultural sites, attending live performances, participating in arts activities and being a member of creative group) [8–15]. The observed social gradient bears resemblance to the gradient seen in health, where privileged individuals are more likely to enjoy better health outcomes [16, 17]. While both arts engagement and health outcomes can be considered ‘assets’ enjoyed by people with more resources, including financial and social support, such engagement can also be seen as a modifiable health behavior that supports and improves health. Indeed, existing literature has shown that arts and cultural engagement is associated with positive health outcomes independent of demographics and socio-economic position [3]. Intervention studies of arts in health have suggested that this may be causal [3]. Differential access to and participation in the arts is therefore a cause for concern as it may be caused by and exacerbate social and health inequalities. However, most of these studies on participation profiles have been limited to Western countries. It remains unclear whether such social patterning of engagement is similar globally and whether inequalities are present internationally, particularly in non-Western countries. Identifying predictors of arts engagement is crucial for designing effective international cultural interventions and policies to promote arts participation and ensure equitable access to cultural opportunities en masse.

This study provides novel evidence on the prevalence of, enablers of, and barriers to arts engagement amongst adults across different cultural settings globally. We analysed data from the World Values Survey (WVS) [18]—a worldwide, cross-sectional dataset—to explore global patterns of arts engagement and identify a range of factors contributing to arts engagement, measured at the country and individual levels. Given arts and cultural engagement is a complex, multifaceted human behavior, we did not attempt to compare all types of such behavior; instead, we focused on one specific form of engagement: participation in arts organizations. Arts organizations are often community-based and can have different manifestations to fit the cultural and social context. Previous literature has suggested that participation in arts and cultural activities in groups can have additional health benefits through social contacts, connections, and interactions (and consequently improve individual and societal wellbeing) [3]. In addition, participation in arts organizations may be more susceptible to social barriers to entry than day-to-day arts engagement. Findings from this study have the potential to guide local, national, and international bodies to formulate public health interventions and policies to increase access to the arts as a strategy for improving population health.

## Methods

### Data

Data from Wave 7 of the WVS were analysed [19]. The WVS is a nationally representative, repeated cross-sectional survey, which studies global variations in participants’ values, attitudes, behaviors, and subjective wellbeing in almost 100 countries. We selected Wave 7 as the most recent wave of data. The wave was open between 2017 and 2023, with most surveys completed in 2018–2020. For more details about the survey, see Additional file 1: Supplementary methods.

In the dataset, 96,006 individuals answered the question on arts organization membership, and 90,890 completed measures of individual socio-demographic variables. Fifteen countries did not include measures on the country-level predictors we planned to include, providing a final analytical sample of 73,825 participants from 51 countries (Additional file 1: Fig. S1). These countries were diverse geographically and economically and included Argentina, Armenia, Australia, Bangladesh, Bolivia, Brazil, Canada, Chile, China, Colombia, Cyprus, Czechia, Ecuador, Egypt, Ethiopia, Germany, Greece, Great Britain, Guatemala, India, Indonesia, Iran, Iraq, Japan, Kazakhstan, Kenya, Kyrgyzstan, Lebanon, Malaysia, Mexico, Mongolia, Morocco, Myanmar, Netherlands, Nicaragua, Pakistan, Peru, Philippines, Romania, Russia, Serbia, South Korea, Tajikistan, Thailand, Tunisia, Turkey, Ukraine, USA, Uruguay, Vietnam, and Zimbabwe.

To explore the rate of arts organization membership across countries, we also examined countries with data on arts organization membership over the last 40 years from 1981 onwards, when the first WVS was conducted, and had at least two waves of data. Data from the WVS time-series dataset were analysed, which combined aggregated data from WVS surveys completed in Waves 1 (1981–1983), 2 (1990–1992), 3 (1995–1998), 4 (2000–2004), 5 (2005–2008), 6 (2010–2014), and 7 (2017–2022) (*N* = 294,243 participants from 69 countries).

### Measures

In the WVS Wave 7, respondents were asked for their membership participation in a list of voluntary organizations such as church or religious organization and environmental organizations, and whether or not they were an “active member”, an “inactive member” or “not a member of that type of organization”. We focused on the item asking for their membership in “art, music or educational organization”, and created a binary indicator for those who reported being a member (either active or inactive) vs those who were not. Although the question includes education alongside arts and music, without specification as to whether that education is related to the arts or broader self-development, the prompt for the question asks people to focus on “voluntary organizations” they are a member of, indicating that this is not a question about participation in formal educational courses.

To understand how organizational membership (hereafter referred to as “arts organization” for brevity) varied across personal characteristics and backgrounds, we considered a rich set of individual-level demographic and socio-economic factors. These included age, gender, country of birth, education level using the United Nations (UN) international standard classification, employment status, and subjective household income. For more details about covariate coding, see Additional file 1: Supplementary measures.

We also considered seven country-level socio-demographic and political factors that were provided in the WVS data. These included (1) life expectancy at birth measured by the World Bank in 2018; (2) proportion of seats held by women in national parliaments measured by the World Bank in 2019; (3) net migration rate measured by the UN Department of Economic and Social Affairs in 2020; (4) compulsory education duration (in years) measured by the World Bank in 2018; (5) unemployment rate (% of total labor force) measured by the World Bank in 2019; (6) Gini income inequality index measured by the World Bank in 2012–2019; and (7) levels of institutionalized democracy measured by Polity in 2018.

### Analysis

To explore the associations between individual-level and country-level predictors and arts organization membership, we used multilevel logistic regression modelling (MLM). We fitted a two-level model to account for the nested structure of the WVS data, as individuals living in the same country might be more similar to each other than to those living in another country. We included country as the higher level to reflect our central hypothesis that variation in arts organization membership may lie at the national level. In the model, we allowed for the estimation of a random slope for gender and age as we assumed that the relationship between gender and age and arts organization membership may vary across countries [9, 11, 12, 15]. This is supported by model fit statistics, which indicated a modest improvement in model fit when random slopes for the two demographic variables were included (Akaike Information Criterion [AIC] without random slopes = 43,771.1; AIC with random slopes = 43,695.37). We also specified an unstructured covariance matrix for the random effects, allowing the variances and covariances among the random effects (for gender and age in this case) to be estimated freely. Three sensitivity analyses were conducted. First, we respecified the outcome variable that compared respondents who were active members with those who were inactive or not members of an arts organization to check whether results were consistent. Second, as the data collection period for WVS Wave 7 spanned across 6 years, we ran an additional parallel analysis, including the survey year as a covariate in the MLM as a robustness check. Third, we used the original arts organization measure and applied ordinal logistic regression within a multilevel model as a robustness check.

Given that aggregating analyses on a global scale may potentially underestimate the effects of individual predictors in different regions, we also ran models independently for seven regions using World Bank groupings: [20] North America (*N* = 6459), Europe and Central Asia (*N* = 20,054), East Asia and Pacific (*N* = 18,033), Latin America and Caribbean (*N* = 14,261), Sub-Saharan Africa (*N* = 3589), Middle East and North Africa (*N* = 7274), and South Asia (*N* = 4155). As some countries had no or very few respondents who were immigrants, country of birth was not included in these regional analyses. Also, as there were few regions overall, MLM was not suitable. We therefore used multivariable logistic regression, testing the associations between individual-level predictors and arts organizational membership separately in each region. We adjusted for country fixed effects in the regression to account for the varying demographics and cultural diversity across countries.

We used odds ratios (OR) and 95% confidence intervals (CIs) to present the associations between individual- and country-level factors and arts organization membership. We applied the population scaled weight, provided in the WVS, to transform each country’s sample size to 1000, so that all countries contributed equally to the combined analysis, while also ensuring the sample size for each country was in proportion to the population size of the region covered by the sample [21]. Missing data were very low (5.3% of participants did not have complete individual socio-economic data) so were handled using listwise deletion. The risk of multicollinearity was minimal (mean VIF = 1.43).

## Results

In our analytical sample, the mean age of respondents was 42.6 (standard deviation [SD] = 16.3; range = 18–89). Participants were older in Japan (mean = 55.1; SD = 17.1), the Netherlands (mean = 54.1; SD = 16.0) and Australia (mean = 53.6; SD = 16.9) and younger in Kenya (mean = 30.8; SD = 10.1), Ethiopia (mean = 31.8; SD = 11.6), and Guatemala (mean = 33.4; SD = 14.1). Generally, 52.7% of our sample were female, 96.9% were native born in their country, 23.2% had a degree, and 58.4% were employed or self-employed (Additional file 1: Tables S1–S6).

### Prevalence of belonging to an arts organization

**Fig. 1:**
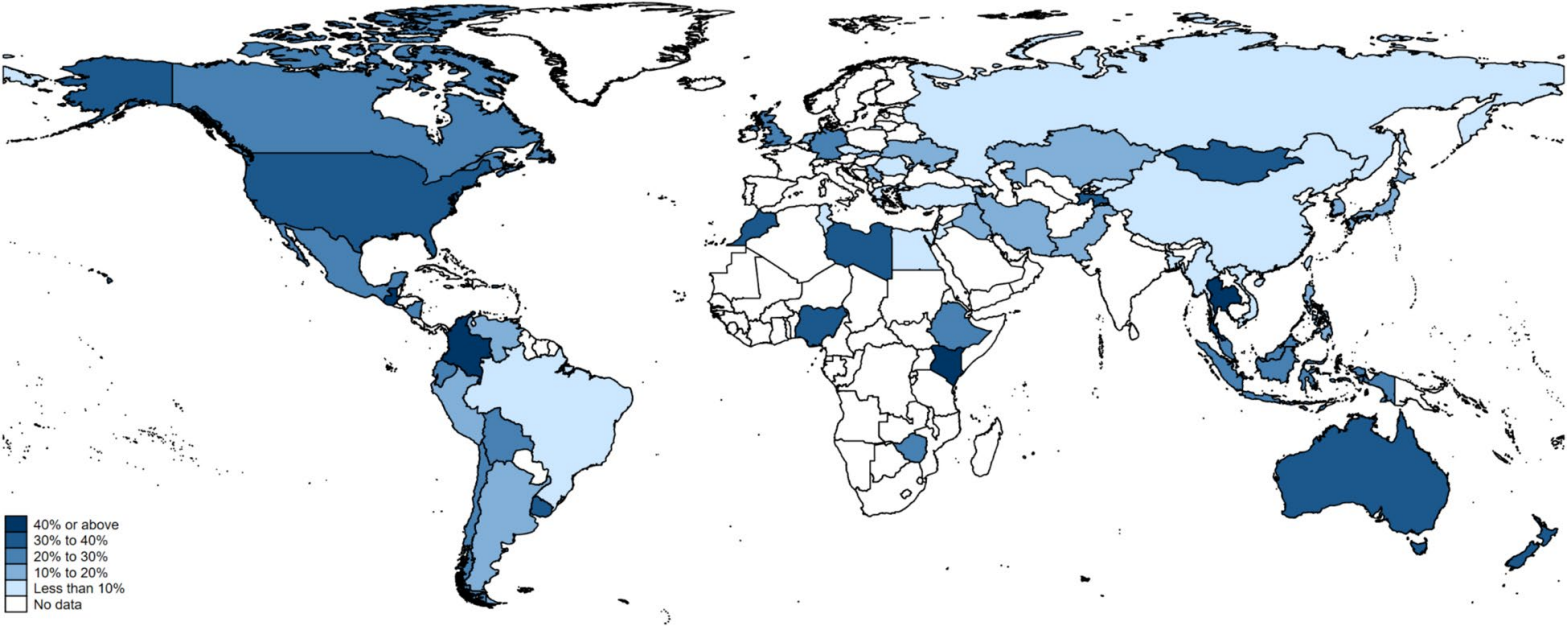
Prevalence of arts organization membership by country in Wave 7 (2017–2022). *N* = 96,006 participants from 66 nations. This map includes all countries with data on arts organization membership Map Source: The World Bank (https://datacatalog.worldbank.org/search/dataset/0038272/World-Bank-Official-Boundaries). *For some countries/regions, there are slight discrepancies between the World Bank map data and data collected in the World Values Survey: Taiwan ROC and Northern Ireland are not shown on the map because they are not listed as independent entities in the World Bank map dataset*

On average, in Wave 7, Kenya (53.7%), Uzbekistan (47.7%), Colombia (44.2%), India and Guatemala (both 42.3%) had the greatest proportion of people reporting membership at an arts organization, followed by Thailand (41.6%), Mongolia (39.6%), Nigeria and Libya (both 38.8%). Vietnam (5.2%), Jordan (4.8%), Czechia (4.6%), and Egypt (3.2%) had the lowest proportion (Fig. 1 and Additional file 1: Table S7). In general, countries with a higher membership rate in arts organizations tend to have more individuals actively involved in these organizations, such as Thailand (27.3%) and Nigeria (24.3%) (Additional file 1: Fig. S2 and Table S7). However, there are some exceptions. For example, in Zimbabwe, while 29.6% reported being a member at an arts organization, only 7.2% were actively involved in it.

The time trends in arts organization membership varied across countries. For instance, this prevalence has remained stable between the 1990s and 2010s in countries such as China, Turkey, and Egypt, while others such as South Korea, Colombia, the USA, and Mexico show a fluctuating trend between 1980/90 s and 2010s. Countries like Uruguay and Cyprus display an increasing trend in the past decade, while Peru and New Zealand demonstrate a decreasing trend (Additional file 1: Fig. S3).

### Individual-level predictors

**Fig. 2:**
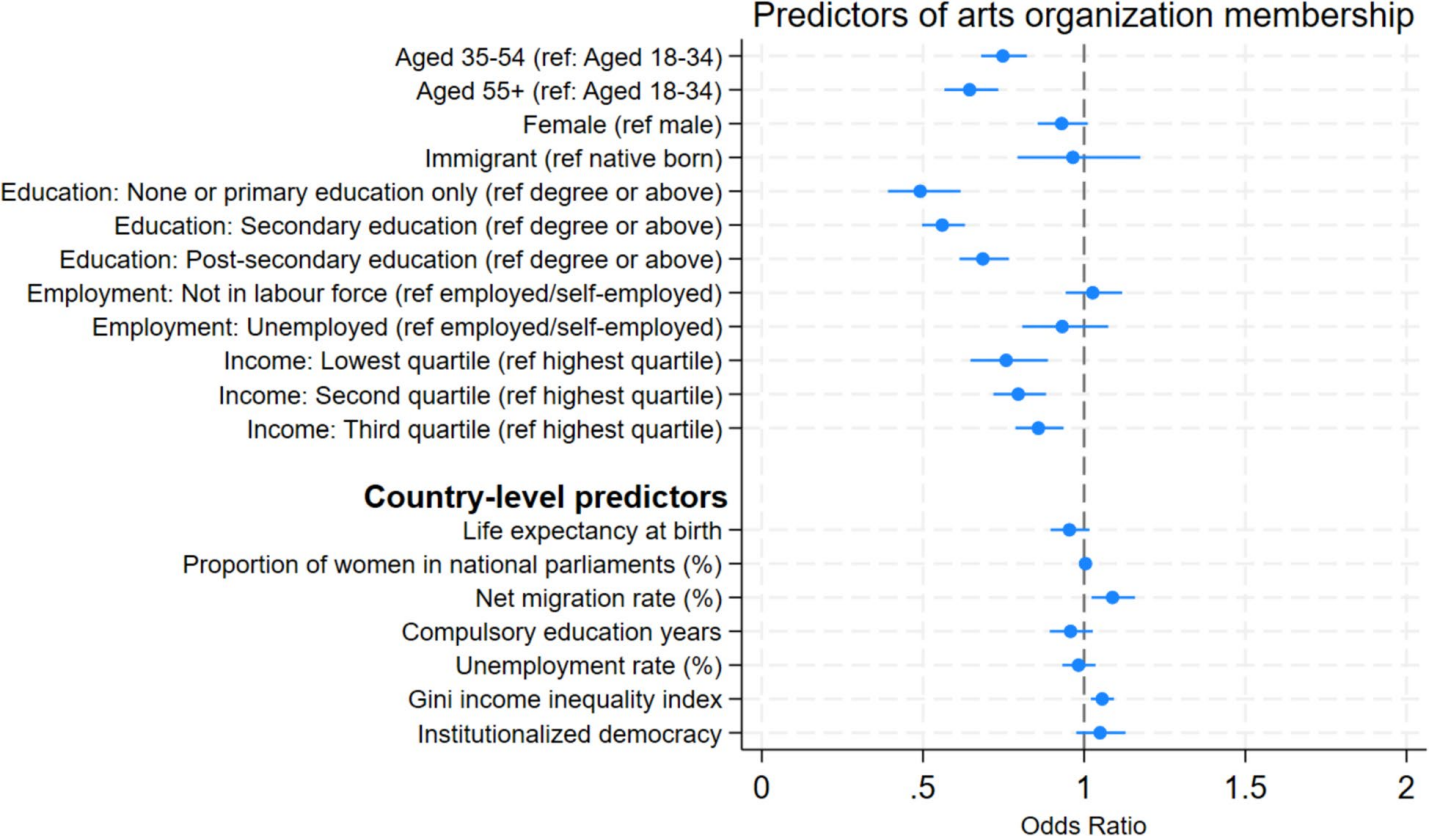
Odds ratios of being in arts organization membership associated with individual- and country-level factors across 51 countries (*N* = 73,825)

Our analysis shows evidence of an individual-level social gradient in arts engagement (Fig. 2; Additional file 1: Table S8). Older adults were less likely to be members of such organizations. Individuals aged 35–54 and those aged 55 or above had 25% lower odds (OR = 0.75, 95%CI = 0.68, 0.82) and 36% lower odds (OR = 0.64, 95%CI 0.57, 0.73) of arts organization membership, respectively, compared to those aged 18–34. However, when zooming in on each individual country, individuals aged 35–54 had higher odds of being members in Peru and Thailand, whereas those aged 55 or over had higher odds in Japan and the Netherlands (Additional file 1: Figs. S4a and S4b). Although no association was found for gender when aggregating the data, we found gender differences in some countries. Particularly, in the Netherlands, Japan, and Australia, females had higher odds of being members of arts organizations (Additional file 1: Fig. S5). South Korea, Lebanon, and Romania show no or little gender differences. Regarding socio-economic status, compared to those with a degree or above, people with no or only primary education had 51% lower odds of arts organization membership (OR = 0.49, 95%CI = 0.39, 0.62), whereas those with secondary education had 44% lower odds (OR = 0.56, 95%CI = 0.50, 0.63) and those with post-secondary education had 31% lower odds (OR = 0.69, 95%CI = 0.61, 0.77). For income, adults who were in the lowest income quartile had the lowest odds of being in an arts organization (lowest quartile: OR = 0.76, 95%CI = 0.65, 0.89; second quartile: OR = 0.80, 95%CI = 0.72, 0.88; third quartile: OR = 0.86, 95%CI = 0.79, 0.94). No associations were found for country of birth or employment status. Sensitivity analysis comparing active membership with inactive or no membership was nearly identical (Additional file 1: Fig. S6). Including the survey year as a covariate or running an ordinal logistic regression within the multilevel modelling did not change the results (Additional file 1: Fig. S7 and Fig. S8).

**Fig. 3:**
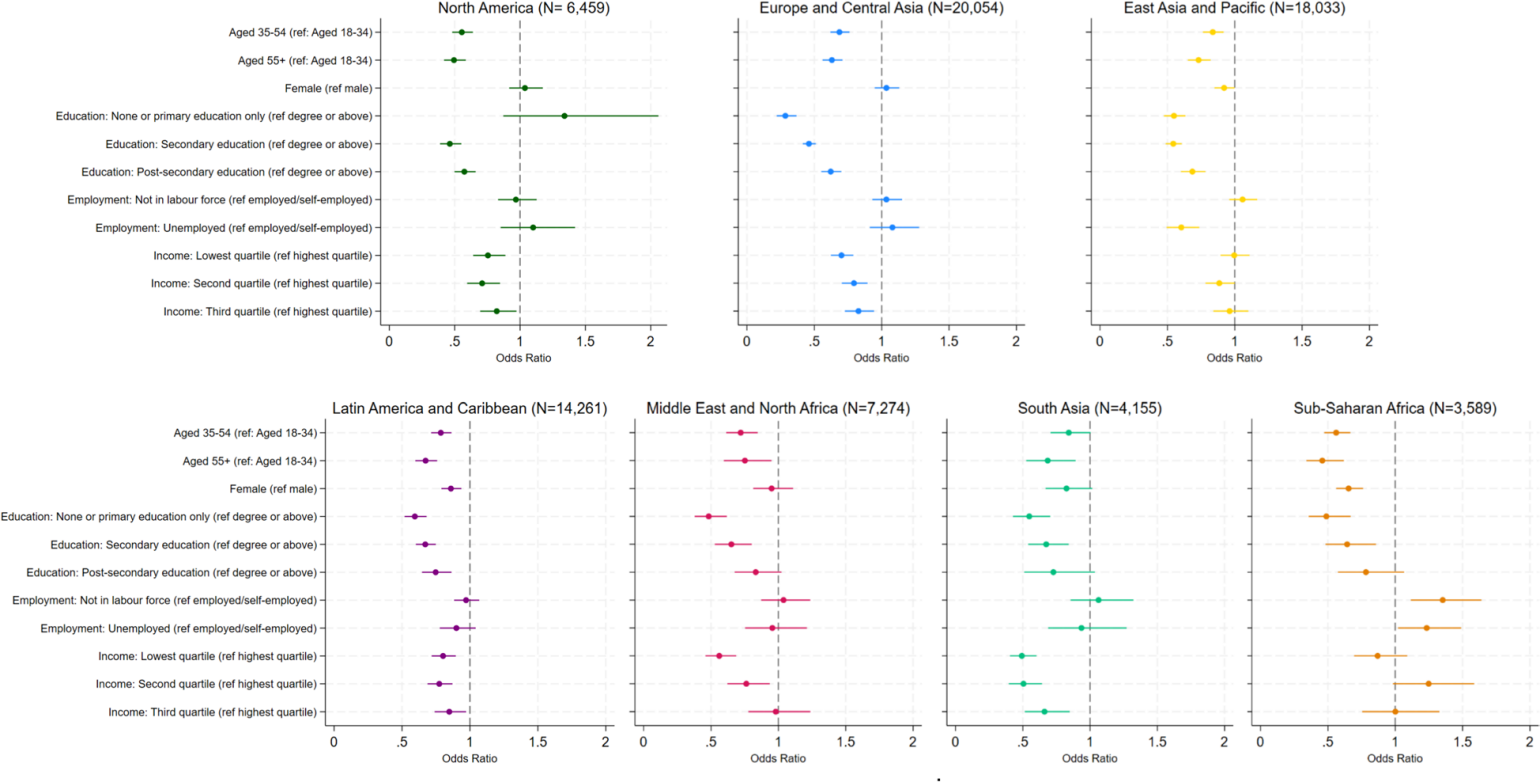
Odds ratios of being in arts organization membership associated with individual-level factors, separately by region. North America includes Canada and the United States. Europe and Central Asia includes Armenia, Cyprus, Czechia, Germany, Greece, Kazakhstan, Kyrgyzstan, Netherlands, Romania, Russia, Serbia, Tajikistan, Turkey, Ukraine, and Great Britain. East Asia and Pacific includes Australia, Myanmar, China, Indonesia, Japan, South Korea, Malaysia, Mongolia, Philippines, Vietnam, and Thailand. Latin America and Caribbean includes Argentina, Bolivia, Brazil, Chile, Colombia, Ecuador, Guatemala, Mexico, Nicaragua, Peru, and Uruguay. Middle East and North Africa includes Iran, Iraq, Lebanon, Morocco, Tunisia, and Egypt. South Asia includes Bangladesh, India, Pakistan. Sub-Saharan Africa includes Ethiopia, Kenya, and Zimbabwe

When dividing the world into regions, we found some consistency in individual-level predictors (Fig. 3; Additional file 1: Table S9). For example, older adults aged 35 or above were less likely than younger adults aged 18 to 34 to be in an arts organization in all regions (ORs ranged from 0.46 to 0.84). Similarly, for education, people with higher education levels were more likely to belong to an arts organization. This educational gradient was most pronounced in Europe and Central Asia, where people with no or primary education or with secondary education had 72% (OR = 0.28, 95%CI = 0.22, 0.37) and 54% (OR = 0.46, 95%CI = 0.41, 0.51) lower odds of being in an arts organization respectively, compared to those with a degree. Income showed a similar pattern but appeared to be less relevant in East Asia and Pacific and in Sub-Saharan African regions. Gender and employment status showed a less consistent pattern. Females had 8% (OR = 0.92, 95%CI = 0.85, 1.00), 14% (OR = 0.86, 95%CI = 0.79, 0.94) and 35% (OR = 0.65, 95%CI = 0.56, 0.76) lower odds of being in an arts organization in East Asia and Pacific, Latin America and Caribbean, and Sub-Saharan Africa, respectively. No gender differences were found in other regions. For employment status, people had 35% (OR = 1.35, 95%CI = 1.12, 1.64) higher odds of being in an arts organization if they were not in the labor force and 23% higher odds (OR = 1.23, 95%CI = 1.02, 1.49) if unemployed in Sub-Saharan Africa, compared to those who were employed/self-employed. However, unemployed participants had 40% (OR = 0.60, 95%CI = 0.49, 0.74) lower odds of arts organization membership in East Asia and Pacific. In contrast, employment status was not associated with arts organization membership in North America, Europe and Central Asia, Latin America and Caribbean, the Middle East and North Africa, or in South Asia.

### Country-level predictors

**Fig. 4:**
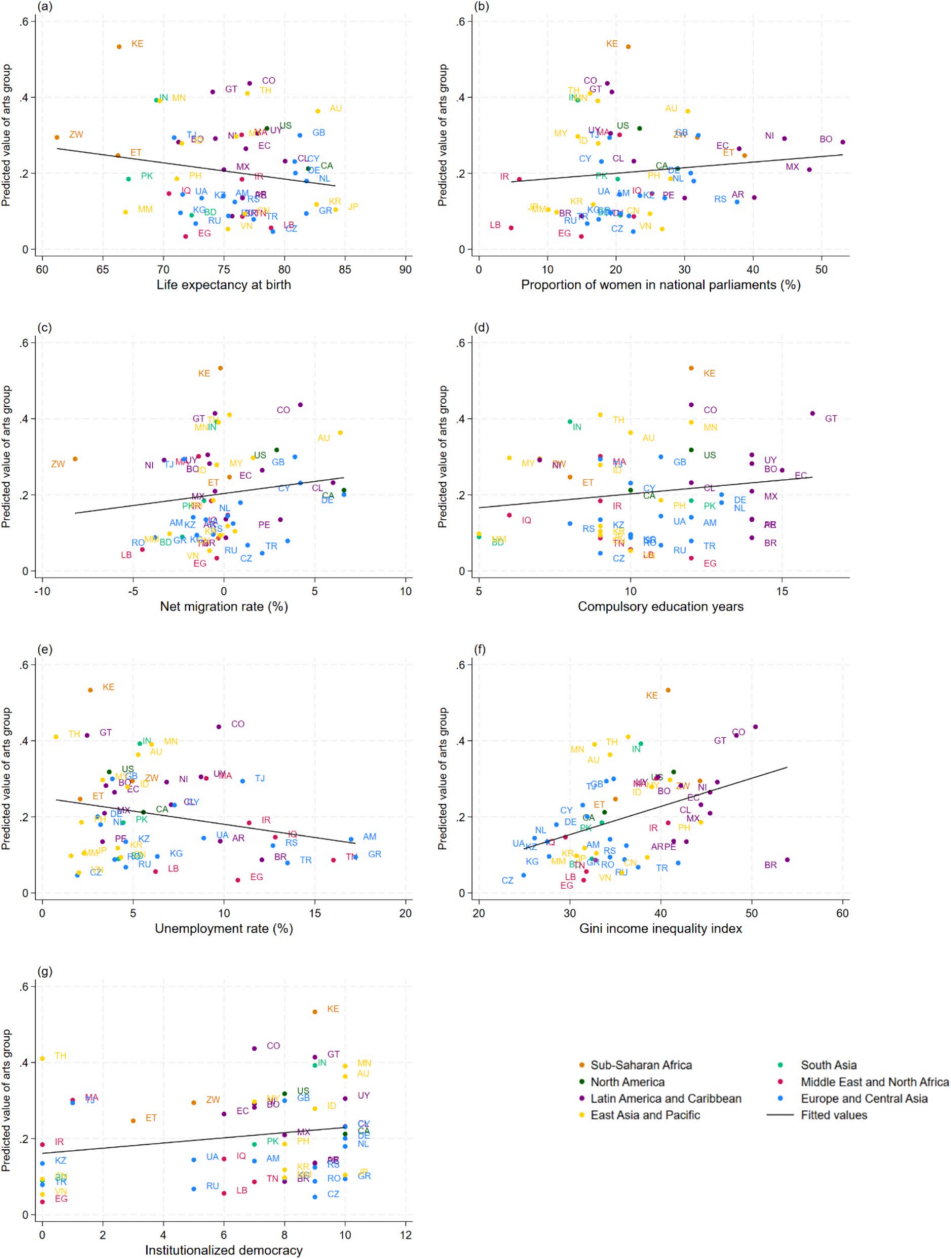
Associations between arts organizationmembership (estimated from multi-level models) and country-level predictors.** a** Life expectancy at birth. **b** Proportion of seats held by women in national parliaments (%). **c** Net migration rate (%). **d** Compulsory education years. **e** Unemployment rate %. **f** Gini income inequality index. **g** Institutionalized democracy. AM = Armenia, AR = Argentina, AU = Australia, BD = Bangladesh, BO = Bolivia, BR = Brazil, CA = Canada, CL = Chile, CN = China, CO = Colombia, CY = Cyprus, CZ = Czechia, DE = Germany, EC = Ecuador, EG = Egypt, ET = Ethiopia, GB = Great Britain, GR = Greece, GT = Guatemala, ID = Indonesia, IN = India, IQ = Iraq, IR = Iran, JP = Japan, KE = Kenya, KG = Kyrgyzstan, KR = South Korea, KZ = Kazakhstan, LB = Lebanon, MA = Morocco, MM = Myanmar, MN = Mongolia, MX = Mexico, MY = Malaysia, NI = Nicaragua, NL = Netherlands, PE = Peru, PH = Philippines, PK = Pakistan, RO = Romania, RS = Serbia, RU = Russia, TH = Thailand, TJ = Tajikistan, TN = Tunisia, TR = Turkey, UA = Ukraine, US = United States, UY = Uruguay, VN = Vietnam, ZW = Zimbabwe

When exploring country-level predictors, net migration rate (OR = 1.09, 95%CI = 1.02, 1.16) and Gini income inequality index (OR = 1.06, 95%CI = 1.02, 1.09) were positively associated with the odds of being in arts organization membership (Fig. 2 and Additional file 1: Table S8). No associations were found for life expectancy at birth, the proportion of seats held by women in national parliaments, years of compulsory education, unemployment rate, and institutionalized democracy. Results were largely consistent in the sensitivity analyses (Additional file 1: Figs. S6–S8). These associations are shown in more detail in Fig. 4, with the predicted probability of arts organization membership from the MLM plotted according to each country-level predictor.

## Discussion

This study explored the prevalence and predictors of arts organization membership across 51 countries in seven regions. The rates vary substantially across countries, from countries where fewer than 1 in 30 people belong to an arts organization (e.g., Egypt) to more than 1 in 2 (e.g., Kenya). Generally, countries with a greater membership rate in arts organizations tend to have more people involved in these organizations actively. Exploring the predictors of organizational membership, we found that factors at both individual and country levels may influence whether or not people belong to an arts organization, with some individual factors persistently being observed across countries and regions while others appearing to be more country- or region-specific.

Our findings are in line with cross-disciplinary evidence that arts organization membership is influenced by individual background and characteristics but also extend the evidence base in several ways. As previously and largely shown in studies from Western countries [9, 11, 13, 15], as well as aligning with epidemiological and social determinants of health and health behaviour literature [16, 17], education plays a significant role in being in arts organization membership. Yet, our analysis also shows that the influence of education can be seen consistently across geographically and culturally diverse regions. Additionally, the educational gradient in arts organization membership is strongest in Europe and Central Asia. Levels of education may contribute to participation in arts organizations in several ways, such as supporting the acquisition of cultural capital and assets (e.g., skills, knowledge, preferences), having higher awareness of activities through social connections, and having higher cognitive capacity to create, interpret and enjoy the arts [22–24]. However, we should consider that our measure of arts organization membership included music, art, and educational organization membership. Given the focus of the question was on voluntary activities (as opposed to formal course engagement), we considered these as related examples of cultural and creative learning, recognizing that learning is a component of arts engagement [2]. Yet, despite this, educational organizations may involve different kinds of behavior to music and art organizations, and may thus be differentially related to education levels. If this was the case, countries with less universal formal education might have higher engagement in voluntary educational groups as proxies for formal educational opportunities. However, we found no evidence that country-level compulsory education was associated with organization membership. This indicates that there is no effect of government education regulations on the membership of organizations discussed in this study over and above individual-level education. Instead, it is possible that other factors such as training in artistic or cultural activity or art education offered in schools may be a more prominent predictor [25].

Subjective household income was another consistent predictor of arts organization membership across countries and regions, a pattern likely driven by economic resources, such as the capacity to pay membership fees [9–11, 14, 15, 25]. This echoes the existing epidemiological literature, which suggests that income plays a crucial role in the systematic inequalities in health [16, 17]. In addition to possessing economic resources, people who perceive their income to be higher may also believe that they have a higher social standing and view arts engagement as a societal expectation that aligns with their perceived status (although this would also depend on culture and cultural values) [23]. This perception could even be greater in countries with more income inequality as, at the country level, higher income inequality (a higher Gini income inequality index) was associated with higher odds of arts organization membership. Yet there are several other potential explanations for this finding, as we do not know whether it is driven by those with lower or higher income. For example, in countries with greater income disparities, arts organizations could be a means for individuals from lower socio-economic backgrounds to access cultural capital, as well as to strengthen social bonds and social capital [26]. Arts organizations could be seen as instruments for national development and social cohesion; hence, participation may be encouraged in countries with greater income inequality [27].

Age, gender, and employment were also associated with arts organization membership. The association between age and arts organization membership was particularly consistent across regions, but the direction of this relationship was in contrast to previous findings [9, 11, 15]. Younger adults were consistently more likely to be members of an arts organization, potentially due to their greater social opportunities. However, there are exceptions. For example, in Japan and the Netherlands, older adults aged 55 + were more likely than individuals aged 18 to 34 to be members of an arts organization. This reflects cultural differences and the possibility that arts organizations may target a more older population demographic in these countries [9, 11, 15, 28] are also some variations in the association between employment and arts organization membership across regions. Particularly, unemployed people living in East Asia and the Pacific had a lower propensity for being members of an arts organization, but this pattern was reversed for people living in Sub-Saharan Africa. This may suggest that people who were unemployed in East Asia and the Pacific experienced greater barriers to arts engagement than people living in Sub-Saharan Africa, potentially due to health conditions, finance, time availability, or local forms of social stigma.

Interestingly, whilst we found that individual-level country of birth was not associated with arts organization membership, country-level net migration was. People were more likely to be arts organization members if they lived in countries with a higher net migration rate (meaning more people immigrating into the country than people leaving that country). This lack of association between individual country of birth and membership might be due to a lack of statistical power, as most participants were native born. However, it may also indicate that society-level immigration is more important than a person’s own country of origin. Countries with a greater net migration rate (often high-income countries) may happen to have more arts and cultural opportunities (e.g., greater private investments in culture through a wider pool of cultural investors) which may help to increase arts organization membership [29]. Alternatively, as these countries are likely to be more ethnically and culturally diverse, more diverse arts organizations may be on offer, and more people may use organizational membership to build their social identity and develop a sense of belonging [26].

The present research has many strengths, including a large sample, nationally representative of 51 countries, enabling us to compare the patterns and predictors of arts organization membership across geographically and culturally diverse regions. The WVS dataset included rich data that allowed us to explore both individual-level and country-level factors. However, the study is not without limitations. First, due to the cross-sectional nature of the data, we were not able to establish causal relationships. Despite this, most of the exposures were likely to temporally precede organizational membership. Our findings provide information that can be used to generate future hypotheses on barriers and facilitators of engagement in the arts using theoretically informed frameworks [30]. 

Second, we were limited by the only available measure of arts and cultural participation in WVS, which focussed on membership in music, arts, and education organizations. Whilst this question was standardized across countries, minimizing measurement biases and enabling large-scale data collection, it has two key weaknesses. It specifically focussed on organization membership, meaning other forms of engagement may have been missed, as many people do music, arts, and education individually or in their communities without being a member of an organization, and broader types of cultural practices were not explored. As such, it presents just part of the overall picture of global patterns of access to arts and culture [7]. Further, the question item asked about participation in voluntary education classes alongside music and arts classes. While these activities all share core social, cultural, and developmental ingredients, future research is encouraged that further seeks to disentangle patterns of engagement across more specific types of arts and cultural practices. Additionally, like many survey studies, the self-reported nature of WVS may consist of recall bias and social desirability bias. Third, despite having explored important individual-level and country-level factors, we lacked information on when respondents started their membership and for how long, as well as community-level factors such as availability and accessibility of organizations in the countries. Qualitative and cultural analysis are also encouraged to provide more in-depth data related to cultural differences in “membership” in terms of experience, practice, and valued offering. While the rich data collection in WVS enabled us to explore the association separately by region, some regions, such as Sub-Saharan Africa, only included Ethiopia, Kenya, and Zimbabwe, which might not be representative of the entire region. Finally, we were not able to include data on how high-level cultural infrastructure and policy differed across countries, which may affect membership rates [6]. For instance, a UNESCO report showed that developed countries were more likely to obtain funds for civil society organizations (who manage and contribute activities in the cultural and creative sectors) from membership fees than developing countries, who tend to receive funding from the private sector, multilateral, bilateral, or civil society bodies and UNESCO or other United Nations agencies.^29^ These country-level differences may also affect the individual-level predictors of organizational membership.

## Conclusions

Overall, our findings indicate that there is inequitable access to and participation in arts, music and education organization membership globally. Education, income and age are important and persistent predictors of membership across regions, alongside other region-specific factors such as gender and employment status. We also identified country-level predictors including net migration rate and Gini income inequality, providing valuable insights into the broader societal, economic, political and cultural factors affecting cultural behaviors and consumption on a national scale. This social gradient in participation is concerning given that people with lower arts engagement rates are also more vulnerable to poorer physical and mental health and wellbeing [3, 16, 17]. The similarity in the predictors of arts organization membership and health suggests two scenarios: firstly, arts organization membership may be considered as ‘assets’ obtained by people with financial and cultural resources. Or secondly, arts organization membership is a health-promoting behavior (similar to physical activity and smoking) and hence is a predictor of health [3]. In these contexts, differential participation rates in arts organization membership could be a cause for concern as they could be exacerbating existing social and health inequalities. If this is the case, then equal access to and participation in the arts may have the potential to help reduce health inequalities through narrowing the gap of social and cultural inequalities between advantaged and disadvantaged populations. Moving forward, a more integrated and multisectoral research approach is needed to (i) continuously and universally monitor engagement patterns across countries (e.g., by incorporating arts engagement into the WHO’s Global Health Observatory), (ii) comprehensively map key persistent and country-specific enablers to democratize engagement, and (iii) develop clear assessments of the relationship between equitable engagement and reduction in health inequalities, as well as track progress over time.

## Supplementary Information

Additional file 1: Supplementary methods: Data from Wave 7 of the World Values Survey (WVS) were analysed. Supplementary measures: We considered a rich set of individual-level demographic and socio-economic factors. Table S1: Average age of the analytical sample. Table S2: Proportion of gender of the analytical sample. Table S3: Proportion of country of birth of the analytical sample. Table S4: Proportion of education level of the analytical sample. Table S5: Proportion of employment status of the analytical sample. Table S6: Average income quartile (ranges from 1–4) of the analytical sample. Table S7: Prevalence of arts organization membership by country (in table).Table S8: Multi-level model estimating odds ratios of being in arts organization membership associated with individual- and country-level factors. Table S9: Logistic regression model estimating odds ratios of being in arts organization membership associated with individual-level factors across seven regions. Figure S1: A flowchart of analytical sample. Figure S2: Prevalence of active arts organization membership by country in Wave 7 (2017–2022). Figure S3: Trends in arts organization membership from 1981 to 2022. Figure S4a: Associations between age (aged 35–54 vs aged 18–34) and arts organization membership across countries (estimated from multi-level models). Figure S4b: Associations between (aged 55 or above vs aged 18–34) and arts organization membership across countries (estimated from multi-level models). Figure S5: Associations between gender and arts organization membership across countries (estimated from multi-level models). Figure S6: Odds ratios of being in active arts organization membership associated with individual- and country- level factors across 51 countries (N = 73,825). Figure S7: Odds ratios of being in arts organization membership associated with individual- and country-level factors across 51 countries (N = 73,825): adding the survey year as a covariate to the model. Figure S8: Odds ratios of being in arts organization membership associated with individual- and country-level factors across 51 countries (N = 73,825): adding using ordinal logistic regression within a multilevel model.

## Data Availability

Data are publicly available on the World Values Survey database site: https://www.worldvaluessurvey.org/wvs.jsp. All code used for these analyses is publicly available online: Mak, H.W., Bone, J.K., Noguchi, T., Kim, J., So, R., Walker, E., Smith, C., Bower, M., Botha, F., Sajnani, N., Fietje, N., Fancourt, D. Global inequalities in arts, music or educational organization membership: an epidemiological analysis of 73,825 adults from 51 countries. Github. https://github.com/karenmak1205/Global-inequalities-in-arts-WVS (2025).
