# Supplementary material for: Global inequalities in arts, music or educational organization membership: an epidemiological analysis of 73,825 adults from 51 countries
Source: BMC Glob Public Health. 2025 Aug 8;3:68. doi: 10.1186/s44263-025-00187-1 (PMC12333132; doi:10.1186/s44263-025-00187-1)
Supplement: Supplementary file 1 — Additional file 1: Supplementary methods: Data from Wave 7 of the World Values Survey (WVS) were analysed. Supplementary measures: We considered a rich set of individual-level demographic and socio-economic factors. Table S1: Average age of the analytical sample. Table S2: Proportion of gender of the analytical sample. Table S3: Proportion of country of birth of the analytical sample. Table S4: Proportion of education level of the analytical sample. Table S5: Proportion of employment status of the analytical sample. Table S6: Average income quartile (ranges from 1–4) of the analytical sample. Table S7: Prevalence of arts organization membership by country (in table).Table S8: Multi-level model estimating odds ratios of being in arts organization membership associated with individual- and country-level factors. Table S9: Logistic regression model estimating odds ratios of being in arts organization membership associated with individual-level factors across seven regions. Figure S1: A flowchart of analytical sample. Figure S2: Prevalence of active arts organization membership by country in Wave 7 (2017–2022). Figure S3: Trends in arts organization membership from 1981 to 2022. Figure S4a: Associations between age (aged 35–54 vs aged 18–34) and arts organization membership across countries (estimated from multi-level models). Figure S4b: Associations between (aged 55 or above vs aged 18–34) and arts organization membership across countries (estimated from multi-level models). Figure S5: Associations between gender and arts organization membership across countries (estimated from multi-level models). Figure S6: Odds ratios of being in active arts organization membership associated with individual- and country- level factors across 51 countries (N = 73,825). Figure S7: Odds ratios of being in arts organization membership associated with individual- and country-level factors across 51 countries (N = 73,825): adding the survey year as a covariate to the model. Figure S8: O [file 44263_2025_187_MOESM1_ESM.docx]

**Additional file 1**

Supplementary Methods

Data from Wave 7 of the World Values Survey (WVS) were analysed.^1^ The wave was open between 2017 and 2023, with most surveys completed in 2018-2020. Approximately 12 countries conducted fieldwork after the COVID-19 pandemic outbreak, so data collection was completed in 2022. The last included survey came from India and was completed in July 2023.^1^ In this wave, over 129,000 respondents were interviewed, spanning 78 countries or territories. Geographic coverage has also been expanded to new countries for the first time, such as Greece, Myanmar, Nicaragua, and Tajikistan. All countries surveyed in Wave 7 employed nationwide random probability sampling, recruiting a minimum sample size of 1200. Samples were representative of all people aged 18 and above residing within private households in each country. The national research team obtained as many Primary Sampling Units as possible to facilitate the sampling process and ensure that the sample is representative of the population of each country. Each Primary Sampling Unit had a maximum of 10 respondents. Data were collected during face-to-face interviews at respondents’ homes or place of residence. Responses were recorded in a paper questionnaire or by CAPI (Computer Assisted Personal Interview).^2,3^

1. Haerpfer, C. *et al.* *World Values Survey: Round Seven – Country-Pooled Datafile Version 6.0.* https://www.worldvaluessurvey.org/WVSDocumentationWV7.jsp (2022).

2. WVS. Fieldwork and Sampling. *World Values Survey* https://www.worldvaluessurvey.org/WVSContents.jsp?CMSID=FieldworkSampling&CMSID=FieldworkSampling (2020).

3. WVS. WVS-7 Fieldwork Progress. *World Values Survey* https://www.worldvaluessurvey.org/WVSContents.jsp?CMSID=WVS7PRO&CMSIS7PRO (2020).

Supplementary Measures

We considered a rich set of individual-level demographic and socio-economic factors. These included age (aged 18-34, aged 35-54, aged 55 or over), gender (female vs male), country of birth (native born vs immigrant), education level using the United Nations (UN) international standard classification (none or primary education only, secondary education, post-secondary education, degree or above), employment status (employed/self-employed, not in labour force [including retired or pensioned/homemaker/student], unemployed), and subjective household income. Subjective household income asked people to indicate where they felt their income group was relative to the rest of the population in their country when counting wages, salaries, pensions and other income, on a scale from 1 (lowest group) to 10 (highest group). We categorised responses on this scale into quartiles (1-4, 5, 6, 7-10). While this is a subjective rather than objective measure of wealth, it provided an assessment of participants’ relative affluence and a way of comparing income levels across countries with very different economic profiles.

# **Table S1: Average age of the analytical sample**

|  | **Arts organization** | | **Not in arts organization** | | **Overall** | |
| --- | --- | --- | --- | --- | --- | --- |
|  | **Mean** | **(SD)** | **Mean** | **(SD)** | **Mean** | **(SD)** |
| Japan | 61.7 | (16.6) | 54.4 | (17.0) | 55.1 | (17.1) |
| Netherlands | 56.0 | (17.5) | 53.7 | (15.6) | 54.1 | (16.0) |
| Australia | 51.9 | (17.6) | 54.5 | (16.4) | 53.6 | (16.9) |
| Germany | 51.9 | (17.4) | 50.7 | (18.0) | 50.9 | (17.9) |
| Great Britain | 50.3 | (18.5) | 51.5 | (17.3) | 51.2 | (17.7) |
| Uruguay | 47.2 | (18.2) | 50.6 | (17.7) | 49.5 | (17.9) |
| Greece | 47.0 | (16.0) | 51.3 | (17.9) | 50.9 | (17.8) |
| Ukraine | 45.2 | (15.7) | 48.2 | (16.7) | 47.8 | (16.6) |
| Thailand | 44.9 | (12.3) | 47.0 | (13.6) | 46.1 | (13.1) |
| Czechia | 43.9 | (18.7) | 49.4 | (16.9) | 49.1 | (17.0) |
| South Korea | 43.6 | (14.7) | 45.9 | (15.1) | 45.6 | (15.0) |
| Romania | 43.1 | (19.6) | 48.9 | (17.5) | 48.4 | (17.8) |
| Chile | 42.6 | (15.8) | 46.0 | (15.4) | 45.3 | (15.6) |
| Myanmar | 42.5 | (15.9) | 40.2 | (14.1) | 40.4 | (14.3) |
| Canada | 42.5 | (18.0) | 47.7 | (16.4) | 46.6 | (16.9) |
| Mexico | 41.2 | (17.0) | 43.9 | (16.4) | 43.3 | (16.5) |
| Philippines | 41.0 | (15.9) | 44.3 | (16.0) | 43.7 | (16.1) |
| Brazil | 40.9 | (18.3) | 45.0 | (16.6) | 44.6 | (16.7) |
| Armenia | 40.4 | (16.1) | 50.4 | (16.8) | 49.0 | (17.0) |
| China | 40.4 | (14.5) | 44.8 | (14.2) | 44.4 | (14.3) |
| United States | 40.0 | (15.9) | 45.4 | (16.2) | 43.7 | (16.3) |
| Tajikistan | 39.7 | (16.1) | 41.7 | (14.9) | 41.1 | (15.3) |
| Peru | 39.4 | (12.1) | 40.4 | (16.0) | 40.3 | (15.5) |
| **Overall** | **39.2** | **(16.1)** | **43.4** | **(16.2)** | **42.6** | **(16.3)** |
| India | 38.4 | (14.4) | 41.4 | (15.1) | 40.2 | (14.9) |
| Russia | 38.3 | (16.4) | 46.2 | (17.1) | 45.6 | (17.1) |
| Egypt | 38.3 | (13.7) | 39.6 | (13.4) | 39.5 | (13.4) |
| Serbia | 38.2 | (15.6) | 47.8 | (17.0) | 46.7 | (17.1) |
| Indonesia | 38.1 | (13.3) | 40.8 | (13.5) | 40.1 | (13.5) |
| Tunisia | 37.9 | (14.2) | 43.2 | (15.3) | 42.7 | (15.3) |
| Argentina | 37.6 | (15.0) | 43.7 | (17.8) | 42.9 | (17.6) |
| Kazakhstan | 37.5 | (12.8) | 42.4 | (14.0) | 41.7 | (13.9) |
| Cyprus | 37.4 | (14.3) | 46.9 | (16.0) | 44.7 | (16.1) |
| Colombia | 37.2 | (15.9) | 40.2 | (15.5) | 38.9 | (15.8) |
| Ecuador | 37.0 | (15.4) | 40.2 | (15.6) | 39.4 | (15.6) |
| Lebanon | 36.6 | (13.9) | 41.1 | (15.5) | 40.8 | (15.4) |
| Mongolia | 36.5 | (13.5) | 40.3 | (14.6) | 38.8 | (14.3) |
| Morocco | 36.4 | (13.6) | 37.6 | (13.5) | 37.2 | (13.5) |
| Malaysia | 36.1 | (12.9) | 39.3 | (13.3) | 38.3 | (13.2) |
| Iraq | 35.8 | (13.7) | 36.8 | (13.4) | 36.6 | (13.4) |
| Vietnam | 35.2 | (12.3) | 38.0 | (12.7) | 37.9 | (12.7) |
| Nicaragua | 35.1 | (13.2) | 36.6 | (13.9) | 36.2 | (13.7) |
| Turkey | 34.9 | (12.9) | 39.1 | (12.5) | 38.8 | (12.6) |
| Zimbabwe | 34.8 | (15.2) | 40.8 | (16.4) | 39.0 | (16.3) |
| Kyrgyzstan | 34.6 | (14.0) | 42.2 | (15.0) | 41.5 | (15.1) |
| Iran | 34.4 | (15.3) | 40.4 | (14.1) | 39.2 | (14.5) |
| Bolivia | 34.3 | (14.7) | 39.5 | (15.9) | 38.0 | (15.7) |
| Bangladesh | 34.1 | (12.7) | 36.9 | (13.0) | 36.6 | (13.0) |
| Pakistan | 33.3 | (11.0) | 36.2 | (11.5) | 35.6 | (11.5) |
| Guatemala | 31.1 | (13.2) | 35.1 | (14.6) | 33.4 | (14.1) |
| Kenya | 29.6 | (9.7) | 32.2 | (10.3) | 30.8 | (10.1) |
| Ethiopia | 28.1 | (9.5) | 33.0 | (11.9) | 31.8 | (11.6) |

# **Table S2: Proportion of gender of the analytical sample**

|  | **Arts organization** | | **Not in arts organization** | | **Overall** | |
| --- | --- | --- | --- | --- | --- | --- |
|  | **Male** | **Female** | **Male** | **Female** | **Male** | **Female** |
| Armenia | 31.4% | 68.6% | 31.3% | 68.7% | 31.3% | 68.7% |
| Uruguay | 31.6% | 68.4% | 32.1% | 67.9% | 31.9% | 68.1% |
| Russia | 32.1% | 67.9% | 41.2% | 58.8% | 40.6% | 59.4% |
| Australia | 32.7% | 67.3% | 43.0% | 57.0% | 39.2% | 60.8% |
| Japan | 36.7% | 63.3% | 46.0% | 54.0% | 45.1% | 54.9% |
| Romania | 37.2% | 62.8% | 39.0% | 61.0% | 38.9% | 61.1% |
| Netherlands | 37.7% | 62.3% | 52.1% | 47.9% | 49.5% | 50.5% |
| Great Britain | 38.2% | 61.8% | 45.4% | 54.6% | 43.2% | 56.8% |
| Greece | 38.9% | 61.1% | 47.8% | 52.2% | 47.0% | 53.0% |
| China | 39.5% | 60.5% | 45.8% | 54.2% | 45.2% | 54.8% |
| Kazakhstan | 39.7% | 60.3% | 44.7% | 55.3% | 44.0% | 56.0% |
| Chile | 43.4% | 56.6% | 49.2% | 50.8% | 47.8% | 52.2% |
| Kyrgyzstan | 44.6% | 55.4% | 37.6% | 62.4% | 38.3% | 61.7% |
| Vietnam | 45.2% | 54.8% | 45.4% | 54.6% | 45.4% | 54.6% |
| Argentina | 45.6% | 54.4% | 48.1% | 51.9% | 47.8% | 52.2% |
| Lebanon | 46.3% | 53.7% | 50.2% | 49.8% | 50.0% | 50.0% |
| Iran | 46.9% | 53.1% | 52.4% | 47.6% | 51.4% | 48.6% |
| Ukraine | 47.1% | 52.9% | 39.6% | 60.4% | 40.7% | 59.3% |
| Czechia | 47.2% | 52.8% | 46.2% | 53.8% | 46.2% | 53.8% |
| Turkey | 48.3% | 51.7% | 50.7% | 49.3% | 50.5% | 49.5% |
| Peru | 48.6% | 51.4% | 50.8% | 49.2% | 50.5% | 49.5% |
| Serbia | 48.7% | 51.3% | 46.6% | 53.4% | 46.8% | 53.2% |
| Mongolia | 48.9% | 51.1% | 48.0% | 52.0% | 48.4% | 51.6% |
| Germany | 49.5% | 50.5% | 48.9% | 51.1% | 49.0% | 51.0% |
| Tunisia | 49.5% | 50.5% | 46.9% | 53.1% | 47.1% | 52.9% |
| **Overall** | **49.6%** | **50.4%** | **46.7%** | **53.3%** | **47.3%** | **52.7%** |
| South Korea | 50.0% | 50.0% | 48.6% | 51.4% | 48.8% | 51.2% |
| Thailand | 50.2% | 49.8% | 44.8% | 55.2% | 47.0% | 53.0% |
| Nicaragua | 51.2% | 48.8% | 47.9% | 52.1% | 48.9% | 51.1% |
| Brazil | 51.6% | 48.4% | 45.6% | 54.4% | 46.1% | 53.9% |
| United States | 51.8% | 48.2% | 54.9% | 45.1% | 53.9% | 46.1% |
| Indonesia | 51.9% | 48.1% | 43.0% | 57.0% | 45.5% | 54.5% |
| Morocco | 52.1% | 47.9% | 49.1% | 50.9% | 50.0% | 50.0% |
| Malaysia | 52.2% | 47.8% | 49.1% | 50.9% | 50.0% | 50.0% |
| Colombia | 52.3% | 47.7% | 48.1% | 51.9% | 49.9% | 50.1% |
| Pakistan | 52.5% | 47.5% | 52.3% | 47.7% | 52.4% | 47.6% |
| Canada | 52.9% | 47.1% | 50.9% | 49.1% | 51.3% | 48.7% |
| Bangladesh | 53.3% | 46.7% | 49.1% | 50.9% | 49.4% | 50.6% |
| Guatemala | 53.4% | 46.6% | 43.7% | 56.3% | 47.7% | 52.3% |
| Mexico | 53.4% | 46.6% | 49.7% | 50.3% | 50.4% | 49.6% |
| Cyprus | 53.8% | 46.2% | 46.3% | 53.7% | 48.0% | 52.0% |
| Tajikistan | 53.8% | 46.2% | 47.5% | 52.5% | 49.4% | 50.6% |
| Kenya | 54.3% | 45.7% | 46.7% | 53.3% | 50.8% | 49.2% |
| Iraq | 54.4% | 45.6% | 49.1% | 50.9% | 49.9% | 50.1% |
| Ecuador | 54.6% | 45.4% | 45.4% | 54.6% | 47.9% | 52.1% |
| Zimbabwe | 55.5% | 44.5% | 46.5% | 53.5% | 49.2% | 50.8% |
| Philippines | 56.8% | 43.2% | 48.5% | 51.5% | 50.0% | 50.0% |
| Bolivia | 59.2% | 40.8% | 46.1% | 53.9% | 49.8% | 50.2% |
| Egypt | 60.0% | 40.0% | 51.9% | 48.1% | 52.1% | 47.9% |
| Ethiopia | 60.2% | 39.8% | 47.6% | 52.4% | 50.7% | 49.3% |
| India | 60.5% | 39.5% | 53.9% | 46.1% | 56.5% | 43.5% |
| Myanmar | 65.2% | 34.8% | 48.5% | 51.5% | 50.1% | 49.9% |

# **Table S3: Proportion of country of birth of the analytical sample**

|  | **Arts organization** | | **Not in arts organization** | | **Overall** | |
| --- | --- | --- | --- | --- | --- | --- |
|  | **Native born** | **Immigrant** | **Native born** | **Immigrant** | **Native born** | **Immigrant** |
| Cyprus | 72.1% | 27.9% | 83.4% | 16.6% | 80.8% | 19.2% |
| Canada | 79.5% | 20.5% | 82.8% | 17.2% | 82.1% | 17.9% |
| Great Britain | 86.0% | 14.0% | 87.3% | 12.7% | 86.9% | 13.1% |
| United States | 89.2% | 10.8% | 89.9% | 10.1% | 89.7% | 10.3% |
| Greece | 90.7% | 9.3% | 88.8% | 11.2% | 89.0% | 11.0% |
| Netherlands | 91.5% | 8.5% | 89.0% | 11.0% | 89.5% | 10.5% |
| Serbia | 91.5% | 8.5% | 91.5% | 8.5% | 91.5% | 8.5% |
| Australia | 92.3% | 7.7% | 92.6% | 7.4% | 92.5% | 7.5% |
| Germany | 93.9% | 6.1% | 84.6% | 15.4% | 86.4% | 13.6% |
| Armenia | 95.9% | 4.1% | 93.0% | 7.0% | 93.4% | 6.6% |
| Argentina | 96.0% | 4.0% | 98.2% | 1.8% | 97.9% | 2.1% |
| Uruguay | 96.6% | 3.4% | 97.1% | 2.9% | 97.0% | 3.0% |
| **Overall** | **96.9%** | **3.1%** | **96.9%** | **3.1%** | **96.9%** | **3.1%** |
| Colombia | 97.1% | 2.9% | 97.0% | 3.0% | 97.1% | 2.9% |
| Ukraine | 97.1% | 2.9% | 95.2% | 4.8% | 95.5% | 4.5% |
| Kyrgyzstan | 97.3% | 2.7% | 94.0% | 6.0% | 94.3% | 5.7% |
| Brazil | 97.7% | 2.3% | 99.6% | 0.4% | 99.5% | 0.5% |
| Malaysia | 97.7% | 2.3% | 99.3% | 0.7% | 98.9% | 1.1% |
| Kazakhstan | 98.0% | 2.0% | 94.5% | 5.5% | 95.0% | 5.0% |
| Tunisia | 98.0% | 2.0% | 98.8% | 1.2% | 98.8% | 1.2% |
| Czechia | 98.1% | 1.9% | 96.9% | 3.1% | 97.0% | 3.0% |
| Chile | 98.2% | 1.8% | 98.4% | 1.6% | 98.4% | 1.6% |
| Russia | 98.2% | 1.8% | 96.0% | 4.0% | 96.2% | 3.8% |
| Japan | 98.3% | 1.7% | 99.0% | 1.0% | 99.0% | 1.0% |
| Ecuador | 98.7% | 1.3% | 99.1% | 0.9% | 99.0% | 1.0% |
| Guatemala | 98.9% | 1.1% | 99.7% | 0.3% | 99.4% | 0.6% |
| Zimbabwe | 99.2% | 0.8% | 99.4% | 0.6% | 99.3% | 0.7% |
| Iran | 99.3% | 0.7% | 99.8% | 0.2% | 99.7% | 0.3% |
| South Korea | 99.3% | 0.7% | 98.9% | 1.1% | 99.0% | 1.0% |
| Kenya | 99.5% | 0.5% | 99.6% | 0.4% | 99.6% | 0.4% |
| Pakistan | 99.7% | 0.3% | 99.7% | 0.3% | 99.7% | 0.3% |
| Tajikistan | 99.7% | 0.3% | 99.4% | 0.6% | 99.5% | 0.5% |
| Bolivia | 99.8% | 0.2% | 99.5% | 0.5% | 99.6% | 0.4% |
| Indonesia | 99.9% | 0.1% | 99.9% | 0.1% | 99.9% | 0.1% |
| Bangladesh | 100.0% | 0.0% | 100.0% | 0.0% | 100.0% | 0.0% |
| Myanmar | 100.0% | 0.0% | 100.0% | 0.0% | 100.0% | 0.0% |
| China | 100.0% | 0.0% | 100.0% | 0.0% | 100.0% | 0.0% |
| Ethiopia | 100.0% | 0.0% | 99.8% | 0.2% | 99.8% | 0.2% |
| India | 100.0% | 0.0% | 100.0% | 0.0% | 100.0% | 0.0% |
| Iraq | 100.0% | 0.0% | 100.0% | 0.0% | 100.0% | 0.0% |
| Lebanon | 100.0% | 0.0% | 99.3% | 0.7% | 99.3% | 0.7% |
| Mexico | 100.0% | 0.0% | 100.0% | 0.0% | 100.0% | 0.0% |
| Mongolia | 100.0% | 0.0% | 100.0% | 0.0% | 100.0% | 0.0% |
| Morocco | 100.0% | 0.0% | 100.0% | 0.0% | 100.0% | 0.0% |
| Nicaragua | 100.0% | 0.0% | 99.8% | 0.2% | 99.8% | 0.2% |
| Peru | 100.0% | 0.0% | 99.5% | 0.5% | 99.6% | 0.4% |
| Philippines | 100.0% | 0.0% | 99.8% | 0.2% | 99.8% | 0.2% |
| Romania | 100.0% | 0.0% | 99.8% | 0.2% | 99.8% | 0.2% |
| Vietnam | 100.0% | 0.0% | 100.0% | 0.0% | 100.0% | 0.0% |
| Thailand | 100.0% | 0.0% | 100.0% | 0.0% | 100.0% | 0.0% |
| Turkey | 100.0% | 0.0% | 99.7% | 0.3% | 99.7% | 0.3% |
| Egypt | 100.0% | 0.0% | 99.9% | 0.1% | 99.9% | 0.1% |

# **Table S4: Proportion of education level of the analytical sample**

|  | **Arts organization** | | **Not in arts organization** | | **Overall** | |
| --- | --- | --- | --- | --- | --- | --- |
|  | **No degree** | **Degree** | **No degree** | **Degree** | **No degree** | **Degree** |
| Serbia | 31.6% | 68.4% | 62.5% | 37.5% | 58.7% | 41.3% |
| Netherlands | 32.4% | 67.6% | 55.9% | 44.1% | 51.6% | 48.4% |
| Australia | 37.8% | 62.2% | 63.3% | 36.7% | 54.0% | 46.0% |
| Canada | 39.5% | 60.5% | 56.9% | 43.1% | 53.2% | 46.8% |
| Cyprus | 40.9% | 59.1% | 63.5% | 36.5% | 58.2% | 41.8% |
| Great Britain | 41.6% | 58.4% | 66.7% | 33.3% | 59.1% | 40.9% |
| Guatemala | 44.8% | 55.2% | 51.9% | 48.1% | 48.9% | 51.1% |
| Kazakhstan | 45.0% | 55.0% | 60.3% | 39.7% | 58.2% | 41.8% |
| Ukraine | 45.9% | 54.1% | 68.8% | 31.2% | 65.4% | 34.6% |
| United States | 47.0% | 53.0% | 63.4% | 36.6% | 58.2% | 41.8% |
| Mongolia | 48.9% | 51.1% | 53.5% | 46.5% | 51.7% | 48.3% |
| Russia | 49.1% | 50.9% | 68.2% | 31.8% | 66.9% | 33.1% |
| Lebanon | 52.2% | 47.8% | 68.4% | 31.6% | 67.5% | 32.5% |
| China | 52.9% | 47.1% | 79.7% | 20.3% | 77.2% | 22.8% |
| Kyrgyzstan | 55.4% | 44.6% | 73.2% | 26.8% | 71.5% | 28.5% |
| Japan | 55.8% | 44.2% | 70.8% | 29.2% | 69.3% | 30.7% |
| Armenia | 56.4% | 43.6% | 72.7% | 27.3% | 70.4% | 29.6% |
| Uruguay | 56.8% | 43.2% | 79.3% | 20.7% | 72.4% | 27.6% |
| South Korea | 59.5% | 40.5% | 69.4% | 30.6% | 68.2% | 31.8% |
| Vietnam | 59.7% | 40.3% | 87.9% | 12.1% | 86.4% | 13.6% |
| Germany | 60.0% | 40.0% | 75.8% | 24.2% | 72.6% | 27.4% |
| Egypt | 60.0% | 40.0% | 84.3% | 15.7% | 83.6% | 16.4% |
| Tajikistan | 61.8% | 38.2% | 74.8% | 25.2% | 70.9% | 29.1% |
| Greece | 62.0% | 38.0% | 77.4% | 22.6% | 75.9% | 24.1% |
| India | 62.8% | 37.2% | 77.5% | 22.5% | 71.7% | 28.3% |
| Turkey | 64.0% | 36.0% | 84.3% | 15.7% | 82.7% | 17.3% |
| Bolivia | 66.5% | 33.5% | 77.9% | 22.1% | 74.7% | 25.3% |
| **Overall** | **68.0%** | **32.0%** | **79.1%** | **20.9%** | **76.8%** | **23.2%** |
| Iran | 69.1% | 30.9% | 74.7% | 25.3% | 73.7% | 26.3% |
| Brazil | 71.1% | 28.9% | 84.8% | 15.2% | 83.7% | 16.3% |
| Czechia | 73.6% | 26.4% | 86.3% | 13.7% | 85.7% | 14.3% |
| Nicaragua | 75.0% | 25.0% | 81.0% | 19.0% | 79.2% | 20.8% |
| Iraq | 79.3% | 20.7% | 81.8% | 18.2% | 81.4% | 18.6% |
| Ecuador | 80.5% | 19.5% | 84.9% | 15.1% | 83.7% | 16.3% |
| Philippines | 80.6% | 19.4% | 92.3% | 7.7% | 90.2% | 9.8% |
| Chile | 81.0% | 19.0% | 87.2% | 12.8% | 85.7% | 14.3% |
| Malaysia | 81.6% | 18.4% | 86.2% | 13.8% | 84.8% | 15.2% |
| Tunisia | 81.8% | 18.2% | 91.5% | 8.5% | 90.7% | 9.3% |
| Colombia | 81.9% | 18.1% | 85.9% | 14.1% | 84.1% | 15.9% |
| Bangladesh | 82.9% | 17.1% | 93.0% | 7.0% | 92.1% | 7.9% |
| Romania | 84.0% | 16.0% | 83.4% | 16.6% | 83.4% | 16.6% |
| Myanmar | 84.3% | 15.7% | 90.6% | 9.4% | 90.0% | 10.0% |
| Morocco | 85.2% | 14.8% | 92.6% | 7.4% | 90.3% | 9.7% |
| Mexico | 86.0% | 14.0% | 92.7% | 7.3% | 91.3% | 8.7% |
| Kenya | 86.5% | 13.5% | 88.9% | 11.1% | 87.6% | 12.4% |
| Ethiopia | 86.6% | 13.4% | 94.6% | 5.4% | 92.6% | 7.4% |
| Argentina | 87.2% | 12.8% | 94.2% | 5.8% | 93.2% | 6.8% |
| Indonesia | 87.6% | 12.4% | 94.4% | 5.6% | 92.5% | 7.5% |
| Pakistan | 87.7% | 12.3% | 90.6% | 9.4% | 90.1% | 9.9% |
| Peru | 91.3% | 8.7% | 85.8% | 14.2% | 86.5% | 13.5% |
| Thailand | 92.6% | 7.4% | 86.9% | 13.1% | 89.2% | 10.8% |
| Zimbabwe | 93.5% | 6.5% | 97.1% | 2.9% | 96.1% | 3.9% |

# **Table S5: Proportion of employment status of the analytical sample**

|  | **Arts organization** | | **Not in arts organization** | | **Overall** | |
| --- | --- | --- | --- | --- | --- | --- |
|  | **Not employed/ unemployed** | **Employed/ self-employed** | **Not employed/ unemployed** | **Employed/ self-employed** | **Not employed/ unemployed** | **Employed/ self-employed** |
| Thailand | 13.0% | 87.0% | 23.7% | 76.3% | 19.3% | 80.7% |
| Kyrgyzstan | 13.4% | 86.6% | 16.0% | 84.0% | 15.8% | 84.2% |
| Kazakhstan | 20.5% | 79.5% | 33.7% | 66.3% | 31.9% | 68.1% |
| Malaysia | 22.0% | 78.0% | 27.6% | 72.4% | 25.9% | 74.1% |
| Myanmar | 22.6% | 77.4% | 25.1% | 74.9% | 24.9% | 75.1% |
| Indonesia | 23.5% | 76.5% | 25.4% | 74.6% | 24.8% | 75.2% |
| Vietnam | 25.8% | 74.2% | 19.9% | 80.1% | 20.3% | 79.7% |
| United States | 27.3% | 72.7% | 35.5% | 64.5% | 32.9% | 67.1% |
| Chile | 29.6% | 70.4% | 30.7% | 69.3% | 30.5% | 69.5% |
| Argentina | 30.4% | 69.6% | 38.4% | 61.6% | 37.3% | 62.7% |
| Guatemala | 30.8% | 69.2% | 37.7% | 62.3% | 34.8% | 65.2% |
| Peru | 32.2% | 67.8% | 34.9% | 65.1% | 34.5% | 65.5% |
| China | 32.3% | 67.7% | 36.3% | 63.7% | 35.9% | 64.1% |
| Mongolia | 32.5% | 67.5% | 30.5% | 69.5% | 31.3% | 68.7% |
| Philippines | 32.9% | 67.1% | 42.4% | 57.6% | 40.7% | 59.3% |
| South Korea | 33.8% | 66.2% | 30.8% | 69.2% | 31.2% | 68.8% |
| Lebanon | 34.3% | 65.7% | 34.6% | 65.4% | 34.6% | 65.4% |
| Czechia | 35.8% | 64.2% | 34.8% | 65.2% | 34.9% | 65.1% |
| Cyprus | 36.1% | 63.9% | 41.9% | 58.1% | 40.6% | 59.4% |
| Egypt | 37.1% | 62.9% | 49.9% | 50.1% | 49.5% | 50.5% |
| Great Britain | 38.6% | 61.4% | 40.2% | 59.8% | 39.7% | 60.3% |
| Ethiopia | 38.8% | 61.2% | 37.8% | 62.2% | 38.1% | 61.9% |
| Turkey | 38.8% | 61.2% | 43.7% | 56.3% | 43.3% | 56.7% |
| Ukraine | 39.0% | 61.0% | 46.1% | 53.9% | 45.1% | 54.9% |
| Brazil | 39.1% | 60.9% | 50.6% | 49.4% | 49.6% | 50.4% |
| **Overall** | **39.4%** | **60.6%** | **42.2%** | **57.8%** | **41.6%** | **58.4%** |
| Canada | 39.6% | 60.4% | 42.8% | 57.2% | 42.1% | 57.9% |
| Germany | 39.7% | 60.3% | 43.2% | 56.8% | 42.5% | 57.5% |
| Russia | 40.2% | 59.8% | 40.0% | 60.0% | 40.1% | 59.9% |
| Australia | 40.3% | 59.7% | 44.0% | 56.0% | 42.7% | 57.3% |
| Mexico | 40.5% | 59.5% | 42.4% | 57.6% | 42.0% | 58.0% |
| Bolivia | 41.7% | 58.3% | 51.1% | 48.9% | 48.5% | 51.5% |
| Greece | 41.7% | 58.3% | 54.7% | 45.3% | 53.5% | 46.5% |
| Serbia | 41.9% | 58.1% | 51.9% | 48.1% | 50.6% | 49.4% |
| Netherlands | 42.3% | 57.7% | 35.8% | 64.2% | 37.0% | 63.0% |
| Kenya | 43.3% | 56.7% | 35.3% | 64.7% | 39.6% | 60.4% |
| Morocco | 44.4% | 55.6% | 43.5% | 56.5% | 43.8% | 56.2% |
| Armenia | 44.8% | 55.2% | 60.5% | 39.5% | 58.2% | 41.8% |
| Ecuador | 46.0% | 54.0% | 51.7% | 48.3% | 50.2% | 49.8% |
| India | 47.9% | 52.1% | 53.7% | 46.3% | 51.4% | 48.6% |
| Japan | 48.3% | 51.7% | 35.8% | 64.2% | 37.1% | 62.9% |
| Pakistan | 48.7% | 51.3% | 48.3% | 51.7% | 48.3% | 51.7% |
| Romania | 48.9% | 51.1% | 54.1% | 45.9% | 53.7% | 46.3% |
| Colombia | 49.5% | 50.5% | 44.9% | 55.1% | 46.9% | 53.1% |
| Iraq | 49.7% | 50.3% | 56.9% | 43.1% | 55.8% | 44.2% |
| Uruguay | 52.4% | 47.6% | 57.0% | 43.0% | 55.6% | 44.4% |
| Tunisia | 54.5% | 45.5% | 50.1% | 49.9% | 50.5% | 49.5% |
| Zimbabwe | 54.7% | 45.3% | 53.9% | 46.1% | 54.1% | 45.9% |
| Bangladesh | 56.2% | 43.8% | 56.3% | 43.7% | 56.3% | 43.7% |
| Tajikistan | 56.7% | 43.3% | 60.2% | 39.8% | 59.1% | 40.9% |
| Iran | 60.4% | 39.6% | 61.6% | 38.4% | 61.3% | 38.7% |
| Nicaragua | 60.5% | 39.5% | 59.7% | 40.3% | 59.9% | 40.1% |

# **Table S6: Average income quartile (ranges from 1-4) of the analytical sample**

|  | **Arts organization** | | **Not in arts organization** | | **Overall** | |
| --- | --- | --- | --- | --- | --- | --- |
|  | **Mean** | **(SD)** | **Mean** | **(SD)** | **Mean** | **(SD)** |
| Romania | 3.13 | (1.13) | 2.42 | (1.18) | 2.48 | (1.19) |
| Canada | 2.89 | (1.17) | 2.61 | (1.19) | 2.67 | (1.19) |
| Bangladesh | 2.86 | (1.20) | 2.61 | (1.21) | 2.63 | (1.21) |
| India | 2.85 | (1.30) | 2.36 | (1.27) | 2.55 | (1.30) |
| Guatemala | 2.79 | (1.18) | 2.78 | (1.13) | 2.79 | (1.15) |
| Kazakhstan | 2.71 | (1.12) | 2.56 | (1.10) | 2.58 | (1.10) |
| Mongolia | 2.71 | (1.16) | 2.48 | (1.13) | 2.57 | (1.15) |
| Turkey | 2.69 | (1.17) | 2.39 | (1.18) | 2.42 | (1.19) |
| Serbia | 2.67 | (1.16) | 2.03 | (1.13) | 2.11 | (1.15) |
| Egypt | 2.66 | (0.94) | 2.18 | (0.99) | 2.19 | (0.99) |
| Australia | 2.65 | (1.22) | 2.35 | (1.21) | 2.46 | (1.22) |
| Netherlands | 2.65 | (1.29) | 2.59 | (1.32) | 2.61 | (1.32) |
| Germany | 2.64 | (1.15) | 2.31 | (1.15) | 2.38 | (1.15) |
| Cyprus | 2.63 | (1.12) | 2.25 | (1.14) | 2.34 | (1.14) |
| Great Britain | 2.62 | (1.29) | 2.38 | (1.30) | 2.45 | (1.30) |
| Lebanon | 2.60 | (1.33) | 2.56 | (1.22) | 2.56 | (1.22) |
| Tajikistan | 2.56 | (1.14) | 2.53 | (1.04) | 2.54 | (1.07) |
| Morocco | 2.52 | (1.10) | 2.23 | (1.14) | 2.32 | (1.13) |
| Vietnam | 2.52 | (1.11) | 2.23 | (1.03) | 2.24 | (1.03) |
| Kyrgyzstan | 2.49 | (1.11) | 2.19 | (1.04) | 2.22 | (1.05) |
| Argentina | 2.43 | (1.08) | 2.19 | (1.10) | 2.22 | (1.10) |
| Bolivia | 2.43 | (1.18) | 2.16 | (1.14) | 2.24 | (1.16) |
| Iran | 2.41 | (1.19) | 1.61 | (0.95) | 1.76 | (1.05) |
| Russia | 2.41 | (1.17) | 2.08 | (1.15) | 2.10 | (1.15) |
| Uruguay | 2.38 | (1.20) | 2.28 | (1.18) | 2.31 | (1.18) |
| United States | 2.37 | (1.19) | 2.24 | (1.16) | 2.28 | (1.17) |
| Ukraine | 2.36 | (1.18) | 1.91 | (1.07) | 1.98 | (1.10) |
| **Overall** | **2.34** | **(1.20)** | **2.15** | **(1.15)** | **2.19** | **(1.17)** |
| Armenia | 2.29 | (1.17) | 2.07 | (1.04) | 2.10 | (1.06) |
| Chile | 2.29 | (1.10) | 1.89 | (1.04) | 1.99 | (1.07) |
| Pakistan | 2.29 | (1.20) | 1.9 | (1.11) | 1.98 | (1.14) |
| South Korea | 2.27 | (0.99) | 2.01 | (1.02) | 2.04 | (1.02) |
| Greece | 2.26 | (1.16) | 1.93 | (1.10) | 1.96 | (1.11) |
| Nicaragua | 2.25 | (1.24) | 2.02 | (1.14) | 2.09 | (1.18) |
| Ecuador | 2.24 | (1.20) | 2.13 | (1.14) | 2.16 | (1.16) |
| Myanmar | 2.22 | (1.09) | 2.03 | (1.07) | 2.05 | (1.07) |
| Tunisia | 2.22 | (1.27) | 2.1 | (1.17) | 2.12 | (1.18) |
| Czechia | 2.21 | (1.13) | 2.36 | (1.16) | 2.35 | (1.15) |
| Mexico | 2.18 | (1.28) | 1.89 | (1.16) | 1.95 | (1.19) |
| Colombia | 2.16 | (1.21) | 1.98 | (1.15) | 2.06 | (1.18) |
| Peru | 2.14 | (1.27) | 2.25 | (1.18) | 2.23 | (1.19) |
| Philippines | 2.11 | (1.10) | 1.91 | (1.05) | 1.95 | (1.06) |
| China | 2.10 | (1.06) | 1.78 | (0.97) | 1.81 | (0.98) |
| Kenya | 2.09 | (1.13) | 1.95 | (1.11) | 2.03 | (1.12) |
| Indonesia | 2.07 | (1.20) | 1.92 | (1.12) | 1.97 | (1.15) |
| Ethiopia | 2.06 | (1.03) | 1.99 | (1.11) | 2.01 | (1.09) |
| Malaysia | 1.94 | (1.17) | 1.84 | (1.19) | 1.87 | (1.18) |
| Japan | 1.86 | (1.26) | 1.93 | (1.23) | 1.92 | (1.23) |
| Brazil | 1.81 | (1.08) | 1.75 | (1.03) | 1.76 | (1.03) |
| Iraq | 1.73 | (0.99) | 1.75 | (1.03) | 1.75 | (1.03) |
| Zimbabwe | 1.68 | (0.95) | 1.56 | (0.90) | 1.60 | (0.92) |
| Thailand | 1.61 | (0.94) | 2.27 | (1.17) | 2.00 | (1.12) |

# **Table S7: Prevalence of arts organization membership by country (in table; 96,006 participants from 66 countries)**

|  | **Arts organization** | **Active membership** | **Inactive membership** | **Not in arts organization** |
| --- | --- | --- | --- | --- |
| Kenya | 53.7% | 30.2% | 24.2% | 46.3% |
| Uzbekistan | 47.7% | 27.2% | 20.5% | 52.3% |
| Colombia | 44.2% | 16.9% | 27.3% | 55.8% |
| India | 42.3% | 21.5% | 20.8% | 57.7% |
| Guatemala | 42.3% | 18.1% | 24.8% | 57.7% |
| Thailand | 41.6% | 27.3% | 14.3% | 58.4% |
| Mongolia | 39.6% | 16.2% | 22.7% | 60.4% |
| Nigeria | 38.8% | 24.3% | 14.5% | 61.2% |
| Libya | 38.8% | 21.1% | 17.8% | 61.2% |
| Australia | 36.4% | 22.5% | 13.9% | 63.6% |
| United States | 32.7% | 16.3% | 15.7% | 67.3% |
| Uruguay | 31.6% | 23.0% | 8.6% | 68.4% |
| New Zealand | 31.5% | 18.9% | 11.9% | 68.5% |
| Tajikistan | 30.5% | 18.8% | 11.0% | 69.5% |
| Morocco | 30.5% | 14.3% | 16.2% | 69.5% |
| Great Britain | 30.1% | 18.3% | 11.1% | 69.9% |
| Nicaragua | 29.9% | 11.7% | 18.2% | 70.1% |
| Malaysia | 29.9% | 10.4% | 19.5% | 70.1% |
| Zimbabwe | 29.6% | 7.2% | 22.4% | 70.4% |
| Bolivia | 28.1% | 18.3% | 10.5% | 71.9% |
| Indonesia | 27.9% | 15.6% | 12.3% | 72.1% |
| Taiwan ROC | 27.9% | 9.1% | 18.8% | 72.1% |
| Ecuador | 26.8% | 14.4% | 11.8% | 73.2% |
| Puerto Rico | 26.6% | 14.3% | 11.7% | 73.4% |
| Cyprus | 26.3% | 13.2% | 13.2% | 73.7% |
| Ethiopia | 25.0% | 14.5% | 9.9% | 75.0% |
| Northern Ireland | 24.3% | 13.2% | 11.2% | 75.7% |
| Hong Kong SAR | 23.4% | 7.1% | 15.6% | 76.6% |
| Chile | 22.7% | 7.1% | 15.6% | 77.3% |
| Canada | 21.4% | 10.4% | 11.0% | 78.6% |
| Mexico | 21.4% | 7.8% | 13.6% | 78.6% |
| Germany | 20.3% | 6.5% | 13.7% | 79.7% |
| Pakistan | 18.8% | 7.0% | 12.3% | 81.2% |
| Iran | 18.8% | 9.7% | 9.1% | 81.2% |
| Philippines | 18.8% | 9.1% | 9.7% | 81.2% |
| Netherlands | 18.2% | 11.9% | 6.3% | 81.8% |
| Singapore | 17.6% | 7.2% | 11.1% | 82.4% |
| Venezuela | 15.6% | 6.5% | 9.1% | 84.4% |
| Macau SAR | 15.0% | 2.4% | 12.4% | 85.0% |
| Kazakhstan | 15.0% | 4.6% | 10.2% | 85.0% |
| Armenia | 14.9% | 8.4% | 6.3% | 85.1% |
| Iraq | 14.7% | 8.0% | 7.3% | 85.3% |
| Ukraine | 14.1% | 4.1% | 10.1% | 85.9% |
| Argentina | 13.6% | 6.2% | 7.1% | 86.4% |
| Peru | 13.2% | 8.6% | 5.3% | 86.8% |
| Andorra | 12.3% | 8.4% | 3.8% | 87.7% |
| South Korea | 12.3% | 4.7% | 7.1% | 87.7% |
| Serbia | 12.3% | 4.9% | 7.5% | 87.7% |
| Japan | 10.5% | 6.6% | 3.7% | 89.5% |
| Slovakia | 10.5% | 3.3% | 7.9% | 89.5% |
| Greece | 9.7% | 4.9% | 4.7% | 90.3% |
| Myanmar | 9.7% | 4.5% | 5.0% | 90.3% |
| Romania | 9.3% | 3.7% | 5.7% | 90.7% |
| Kyrgyzstan | 9.2% | 5.6% | 3.9% | 90.8% |
| China | 9.2% | 3.1% | 6.1% | 90.8% |
| Tunisia | 8.7% | 3.4% | 5.4% | 91.3% |
| Brazil | 8.7% | 6.3% | 2.5% | 91.3% |
| Bangladesh | 8.7% | 4.9% | 4.2% | 91.3% |
| Maldives | 8.0% | 2.5% | 5.7% | 92.0% |
| Turkey | 7.9% | 2.2% | 5.7% | 92.1% |
| Russia | 6.6% | 2.8% | 3.8% | 93.4% |
| Lebanon | 5.8% | 2.3% | 3.2% | 94.2% |
| Vietnam | 5.2% | 3.1% | 2.1% | 94.8% |
| Jordan | 4.8% | 3.2% | 2.1% | 95.2% |
| Czechia | 4.6% | 2.6% | 2.1% | 95.4% |
| Egypt | 3.2% | 1.9% | 1.1% | 96.8% |

# **Table S8: Multi-level model estimating odds ratios of being in arts organization membership associated with individual- and country-level factors.**

|  | **OR** | **95%CI** |
| --- | --- | --- |
| **Fixed part** |  |  |
| **Individual-level factors** |  |  |
| Aged 35-54 (ref Aged 18-34) | **0.75** | **0.68, 0.82** |
| Aged 55 or above (ref Aged 18-34) | **0.64** | **0.57, 0.73** |
| Female (ref male) | 0.93 | 0.86, 1.01 |
| Immigrant (ref native born) | 0.97 | 0.79, 1.17 |
| Education: None or primary education only (ref degree or above) | **0.49** | **0.39, 0.62** |
| Education: Secondary education (ref degree or above) | **0.56** | **0.50, 0.63** |
| Education: Post-secondary education (ref degree or above) | **0.69** | **0.61, 0.77** |
| Employment: Not in labour force (ref employed/self-employed) | 1.03 | 0.94, 1.12 |
| Employment: Unemployed (ref employed/self-employed) | 0.93 | 0.81, 1.07 |
| Income: Lowest quartile (ref highest quartile) | **0.76** | **0.65, 0.89** |
| Income: Second quartile (ref highest quartile) | **0.80** | **0.72, 0.88** |
| Income: Third quartile (ref highest quartile) | **0.86** | **0.79, 0.94** |
| **Country-level factors** |  |  |
| Life expectancy at birth | 0.95 | 0.90, 1.02 |
| Proportion of seats held by women in national parliaments (%) | 1.00 | 0.99, 1.02 |
| Net migration rate (%) | **1.09** | **1.02, 1.16** |
| Compulsory education years | 0.96 | 0.89, 1.03 |
| Unemployment rate (%) | 0.98 | 0.93, 1.04 |
| Gini income inequality index | **1.06** | **1.02, 1.09** |
| Institutionalized democracy | 1.06 | 0.98, 1.13 |
| **Random part** |  |  |
| Variation in female | 0.04 | 0.02, 0.09 |
| Variation in aged 35-54 | 0.06 | 0.03, 0.11 |
| Variation in aged 55 or above | 0.13 | 0.06, 0.29 |
| Variation in the intercept | 0.46 | 0.29, 0.73 |
| Co-variance (female, aged 35-54) | 0.01 | -0.01, 0.04 |
| Co-variance (female, aged 55 or above) | 0.03 | -0.01, 0.08 |
| Co-variance (female, constant) | -0.05 | -0.13, 0.02 |
| Co-variance (aged 35-54, aged 55 or above) | 0.07 | 0.03, 0.11 |
| Co-variance (aged 35-54, constant) | -0.03 | -0.11, 0.05 |
| Co-variance (aged 55 or above, constant) | -0.05 | -0.16, 0.06 |
| **Number of observations** | 73,825 |  |
| **Number of countries** | 51 |  |
| Note: Bold values denote statistical significance at the p<0.05 level. | | |

# **Table S9: Logistic regression model estimating odds ratios of being in arts organization membership associated with individual-level factors across seven regions.**

|  | **North America** | | **Europe and Central Asia** | | **East Asia and Pacific** | | **Latin America and Caribbean** | | **Middle East and North Africa** | | **South Asia** | | **Sub Saharan Africa** | |
| --- | --- | --- | --- | --- | --- | --- | --- | --- | --- | --- | --- | --- | --- | --- |
|  | **OR** | **95%CI** | **OR** | **95%CI** | **OR** | **95%CI** | **OR** | **95%CI** | **OR** | **95%CI** | **OR** | **95%CI** | **OR** | **95%CI** |
| Aged 35-54 (ref Aged 18-34) | **0.55** | **0.48, 0.64** | **0.69** | **0.62, 0.76** | **0.84** | **0.76, 0.92** | **0.79** | **0.71, 0.86** | **0.72** | **0.61, 0.85** | 0.84 | 0.71, 1.00 | **0.56** | **0.47, 0.67** |
| Aged 55 or above (ref Aged 18-34) | **0.49** | **0.42, 0.58** | **0.63** | **0.56, 0.71** | **0.73** | **0.65, 0.82** | **0.67** | **0.60, 0.76** | **0.75** | **0.60, 0.95** | **0.68** | **0.53, 0.89** | **0.46** | **0.34, 0.62** |
| Female (ref male) | 1.04 | 0.92, 1.17 | 1.04 | 0.95, 1.13 | **0.92** | **0.85, 1.00** | **0.86** | **0.79, 0.94** | 0.95 | 0.81, 1.11 | 0.82 | 0.67, 1.02 | **0.65** | **0.56, 0.76** |
| Education: None or primary education only (ref degree or above) | 1.34 | 0.87, 2.06 | **0.28** | **0.22, 0.37** | **0.55** | **0.47, 0.63** | **0.59** | **0.52, 0.68** | **0.48** | **0.38, 0.62** | **0.55** | **0.43, 0.70** | **0.49** | **0.36, 0.67** |
| Education: Secondary education (ref degree or above) | **0.46** | **0.39, 0.55** | **0.46** | **0.41, 0.51** | **0.54** | **0.49, 0.61** | **0.67** | **0.60, 0.75** | **0.65** | **0.53, 0.80** | **0.67** | **0.54, 0.84** | **0.64** | **0.48, 0.86** |
| Education: Post-secondary education (ref degree or above) | **0.57** | **0.50, 0.66** | **0.62** | **0.55, 0.70** | **0.69** | **0.60, 0.78** | **0.75** | **0.65, 0.86** | 0.83 | 0.68, 1.02 | 0.73 | 0.51, 1.04 | 0.78 | 0.57, 1.07 |
| Employment: Not in labour force (ref employed/self-employed) | 0.97 | 0.83, 1.13 | 1.04 | 0.93, 1.15 | 1.06 | 0.96, 1.17 | 0.97 | 0.88, 1.07 | 1.04 | 0.87, 1.24 | 1.06 | 0.85, 1.32 | **1.35** | **1.12, 1.64** |
| Employment: Unemployed (ref employed/self-employed) | 1.10 | 0.85, 1.42 | 1.08 | 0.91, 1.28 | **0.60** | **0.49, 0.74** | 0.90 | 0.78, 1.04 | 0.96 | 0.75, 1.21 | 0.94 | 0.69, 1.27 | **1.23** | **1.02, 1.49** |
| Income: Lowest quartile (ref highest quartile) | **0.75** | **0.64, 0.89** | **0.70** | **0.62, 0.79** | 1.00 | 0.89, 1.11 | **0.80** | **0.72, 0.90** | **0.56** | **0.46, 0.69** | **0.49** | **0.40, 0.60** | 0.87 | 0.69, 1.09 |
| Income: Second quartile (ref highest quartile) | **0.71** | **0.60, 0.85** | **0.79** | **0.70, 0.90** | 0.89 | 0.78, 1.00 | **0.77** | **0.69, 0.87** | **0.76** | **0.62, 0.94** | **0.50** | **0.40, 0.64** | 1.25 | 0.98, 1.59 |
| Income: Third quartile (ref highest quartile) | **0.82** | **0.69, 0.97** | **0.83** | **0.73, 0.94** | 0.96 | 0.84, 1.10 | **0.85** | **0.74, 0.97** | 0.98 | 0.78, 1.24 | **0.66** | **0.52, 0.85** | 1.00 | 0.76, 1.33 |
| Constant | **0.64** | **0.55, 0.76** | **0.41** | **0.34, 0.51** | 1.00 | 0.86, 1.18 | **0.36** | **0.29, 0.45** | **0.51** | **0.39, 0.66** | **0.26** | **0.19, 0.35** | **0.71** | **0.51, 0.99** |
| **N** | **6,459** | | **20,054** | | **18,033** | | **14,261** | | **7,274** | | **4,155** | | **3,589** | |
| Note: All models adjusted for country fixed effects to account for the varying demographics and cultural diversity across countries. Bold values denote statistical significance at the p<0.05 level. | | | | | | | | | | | | | | |

Individuals who completed arts organization membership question (N=96,006; 66 countries)

Individuals who completed measures of individual socio-demographic variables (N=90,890; 66 countries)

Countries with complete country-level variables (N=73,825; 51 countries)

N=5,116 Individuals did not complete all measures of individual socio-demographic variables

15 countries (N=17,065) did not have all measures across country-level variables

# **Fig. S1: A flowchart of analytical sample.**


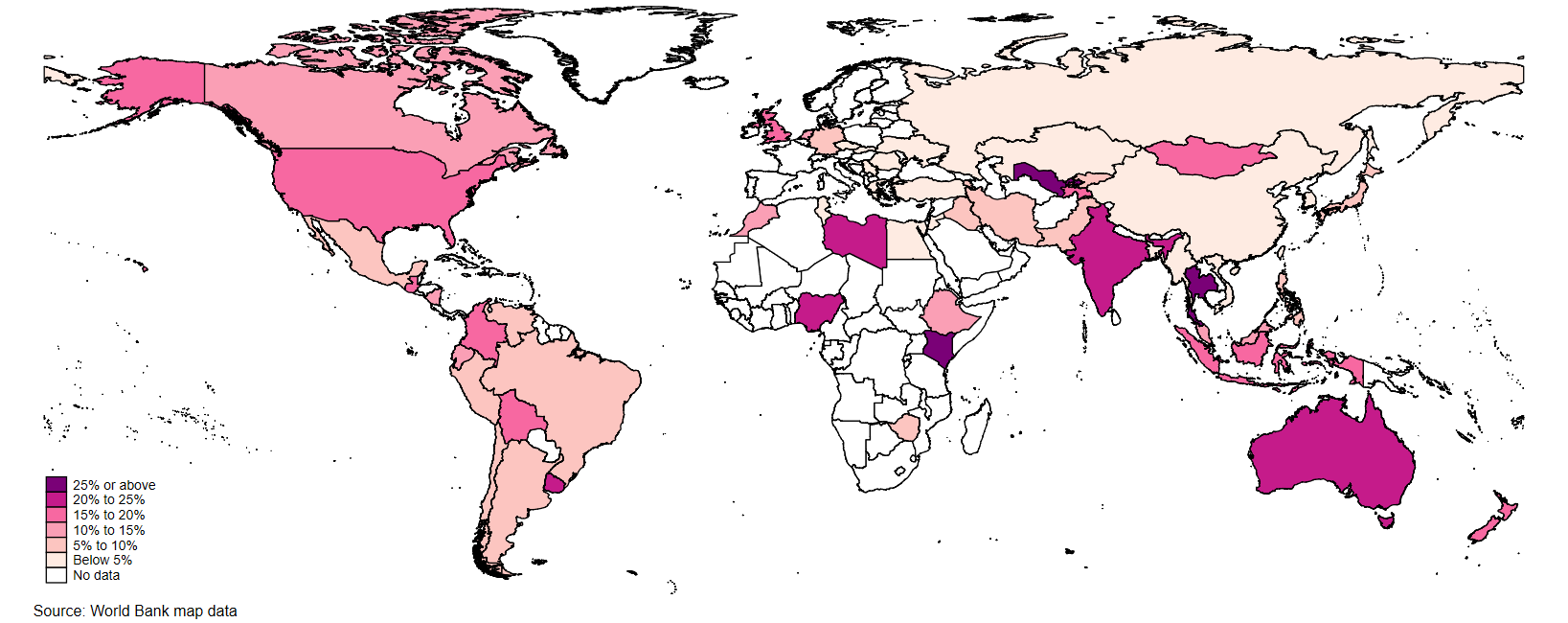


# **Fig. S2: Prevalence of active arts organization membership by country in Wave 7 (2017-2022).** N= 96,006 participants from 66 nations. This map includes all countries with data on arts organization membership. *For some countries/regions, there are slight discrepancies between the World Bank map data and data collected in the World Values Survey: Taiwan ROC and Northern Ireland are not shown on the map because they are not listed as independent entities in the World Bank map dataset.*


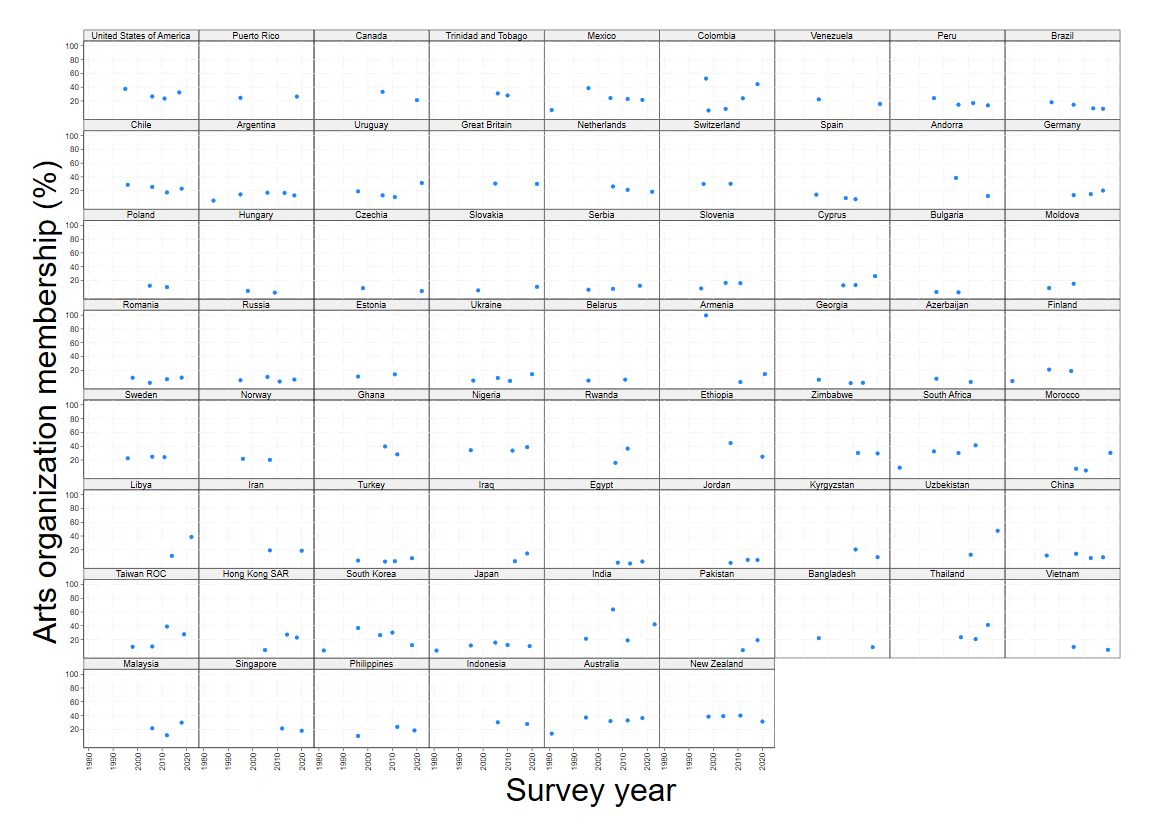


# **Fig. S3: Trends in arts organization membership from 1981 to 2022.** This includes all countries that had historical data on arts organization membership and at least two waves of data (294,243 participants from 69 countries).


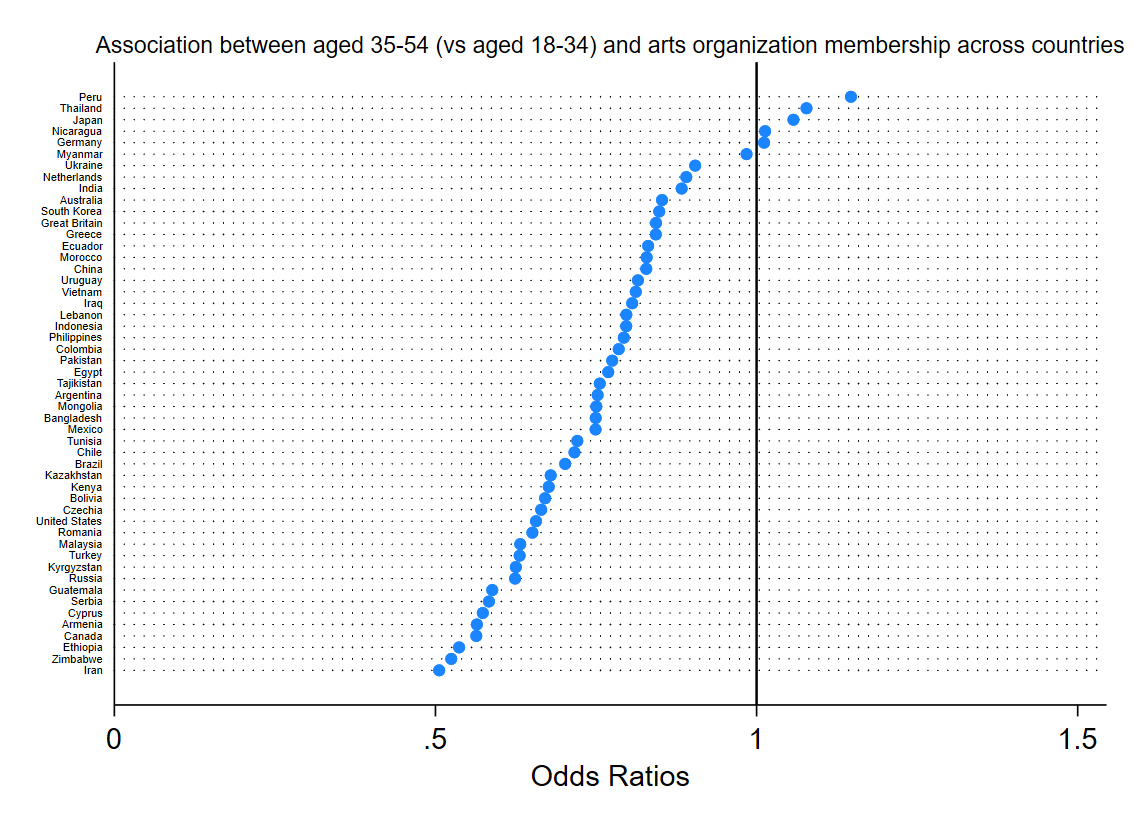
 **Fig. S4a: Associations between age (aged 35-54 vs aged 18-34) and arts organization membership across countries (estimated from multi-level models).** An odds ratio (OR) greater than 1 indicates that those aged 35-54 have higher odds of being members, while an OR less than 1 indicates those aged 18-34 have higher odds of being members.


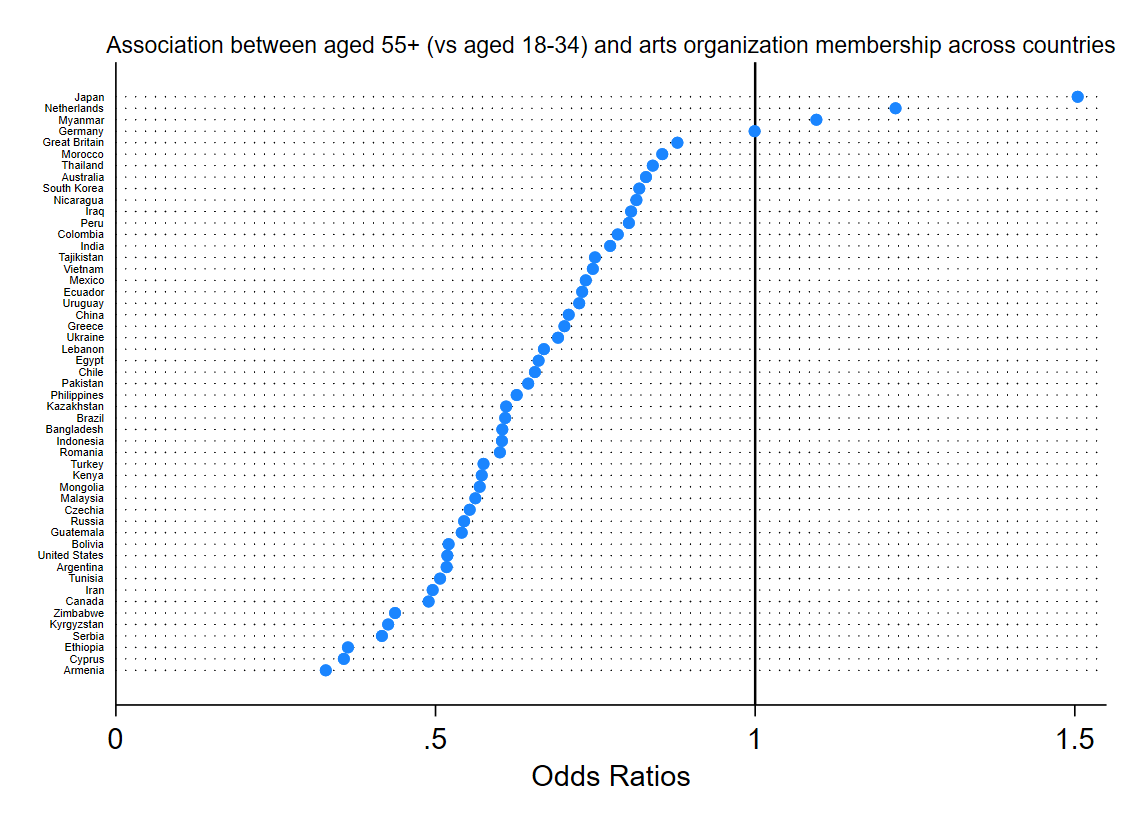


# **Fig. S4b: Associations between (aged 55 or above vs aged 18-34) and arts organization membership across countries (estimated from multi-level models).** An odds ratio (OR) greater than 1 indicates that individuals aged 55+ have higher odds of being members, while an OR less than 1 indicates individuals aged 18-34 have higher odds of being members.


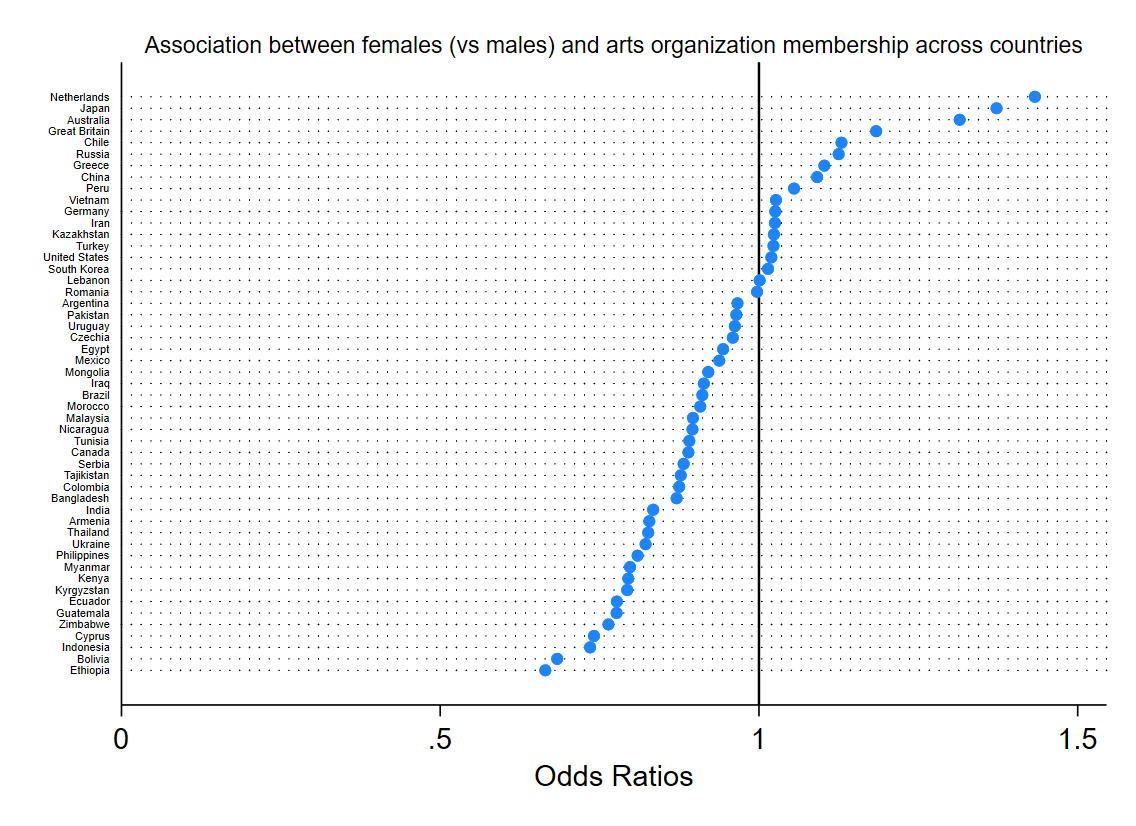


# **Fig. S5: Associations between gender and arts organization membership across countries (estimated from multi-level models).** An odds ratio (OR) greater than 1 indicates that females have higher odds of being members, while an OR less than 1 indicates males have higher odds of being members.

**
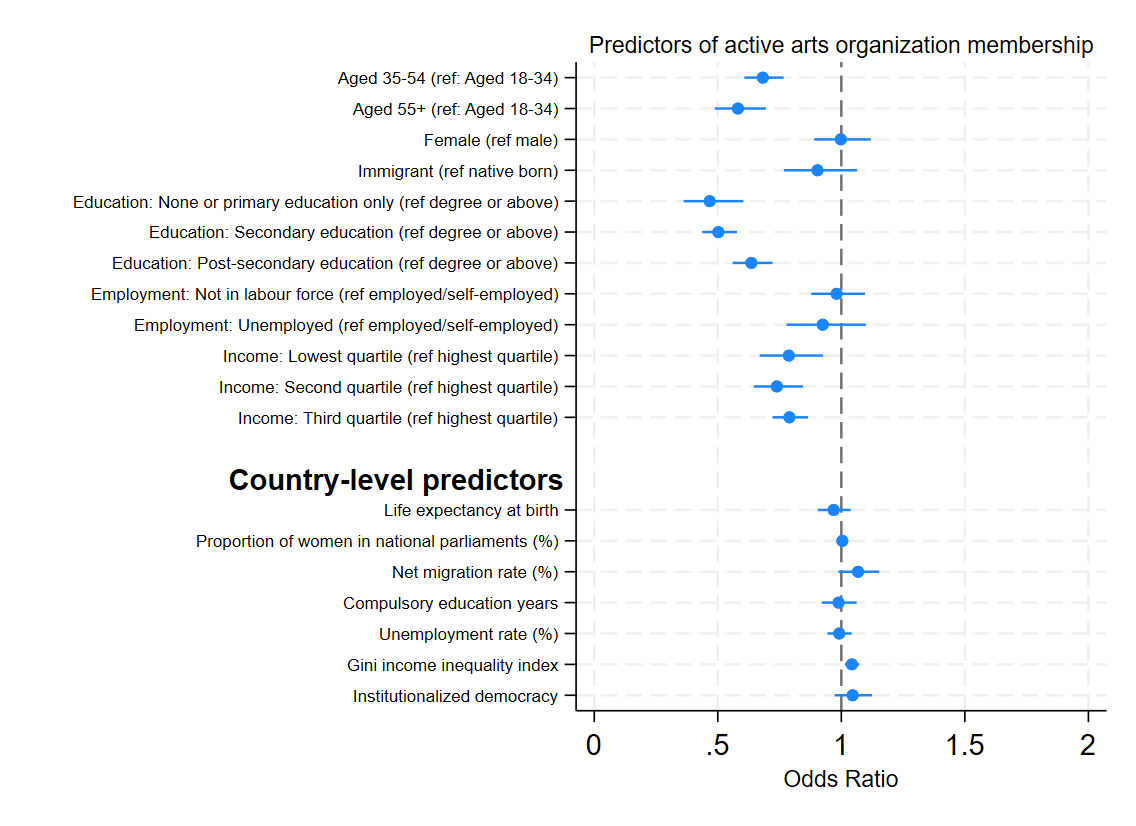
**

# **Fig. S6: Odds ratios of being in *active* arts organization membership associated with individual- and country-level factors across 51 countries (N= 73,825).**


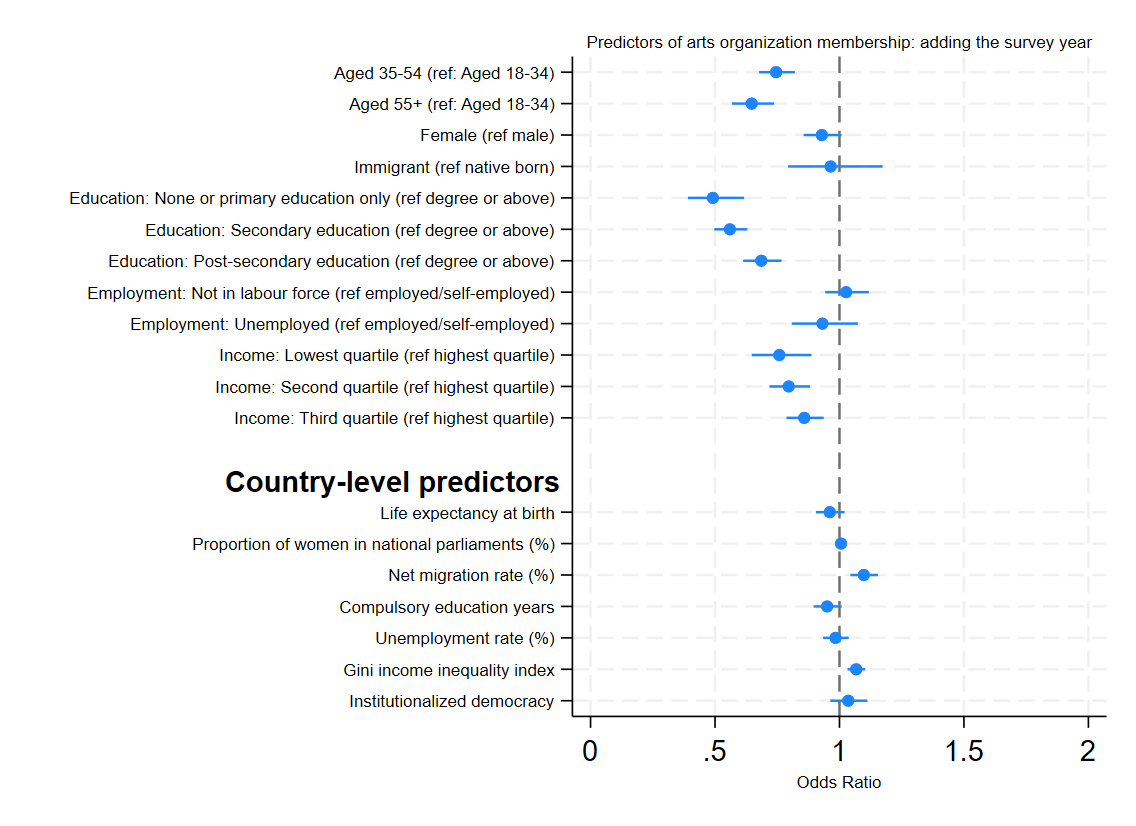


# **Fig. S7: Odds ratios of being in arts organization membership associated with individual- and country-level factors across 51 countries (N= 73,825): adding the survey year as a covariate to the model.**


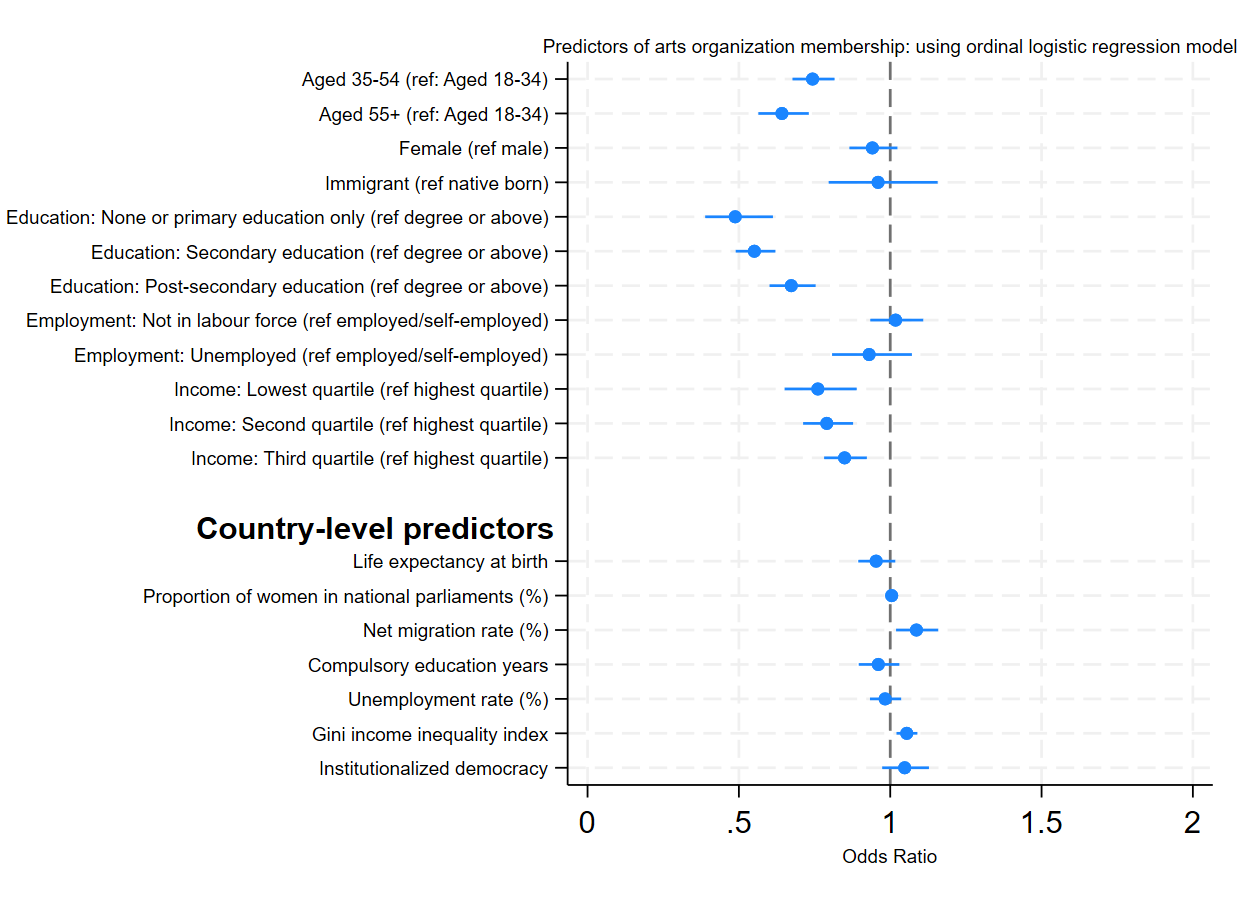


# **Fig. S8: Odds ratios of being in arts organization membership associated with individual- and country-level factors across 51 countries (N= 73,825): adding using ordinal logistic regression within a multilevel model.**
